# Probing the Role of Murine Neuroglobin CDloop–D-Helix Unit in CO Ligand Binding and Structural Dynamics

**DOI:** 10.1021/acschembio.2c00172

**Published:** 2022-07-07

**Authors:** Cécile Exertier, Federico Sebastiani, Ida Freda, Elena Gugole, Gabriele Cerutti, Giacomo Parisi, Linda Celeste Montemiglio, Maurizio Becucci, Cristiano Viappiani, Stefano Bruno, Carmelinda Savino, Carlotta Zamparelli, Massimiliano Anselmi, Stefania Abbruzzetti, Giulietta Smulevich, Beatrice Vallone

**Affiliations:** † Dipartimento di Scienze Biochimiche “A. Rossi Fanelli”, Sapienza, Università di Roma, Piazzale A. Moro 5, I-00185 Rome, Italy; ‡ Dipartimento di Chimica ″Ugo Schiff″, Università di Firenze, Via della Lastruccia 3-13, I-50019 Sesto Fiorentino, Italy; § Zuckerman Mind Brain Behavior Institute, Columbia University, 3227 Broadway, New York, New York 10027, United States; ∥ Center for Life Nanoscience, Istituto Italiano di Tecnologia, Viale Regina Elena, 291, I-00161 Rome, Italy; ⊥ Institute of Molecular Biology and Pathology, National Research Council, Piazzale A. Moro 5, 00185 Rome, Italy; # Department of Mathematical, Physical and Computer Sciences, University of Parma, Parco Area delle Scienze, 7/A, I-43124 Parma, Italy; ∇ Department of Food and Drugs, University of Parma, Parco Area delle Scienze 27/A, I-43124 Parma, Italy; ○ Theoretical Physics and Center for Biophysics, Saarland University, Campus E2 6, 66123 Saarbrücken, Germany

## Abstract

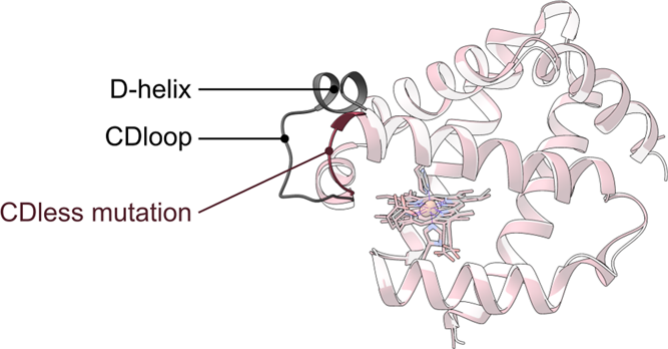

We produced a neuroglobin variant, namely, Ngb CDless, with the excised CDloop- and D-helix, directly joining the C- and E-helices. The CDless variant retained bis-His hexacoordination, and we investigated the role of the CDloop–D-helix unit in controlling the CO binding and structural dynamics by an integrative approach based on X-ray crystallography, rapid mixing, laser flash photolysis, resonance Raman spectroscopy, and molecular dynamics simulations. Rapid mixing and laser flash photolysis showed that ligand affinity was unchanged with respect to the wild-type protein, albeit with increased on and off constants for rate-limiting heme iron hexacoordination by the distal His64. Accordingly, resonance Raman spectroscopy highlighted a more open distal pocket in the CO complex that, in agreement with MD simulations, likely involves His64 swinging inward and outward of the distal heme pocket. Ngb CDless displays a more rigid overall structure with respect to the wild type, abolishing the structural dynamics of the CDloop–D-helix hypothesized to mediate its signaling role, and it retains ligand binding control by distal His64. In conclusion, this mutant may represent a tool to investigate the involvement of CDloop–D-helix in neuroprotective signaling in a cellular or animal model.

## Introduction

Neuroglobin (Ngb) is a phylogenetically ancient representative of the globin superfamily found in vertebrates and predominantly expressed in the central nervous system at overall concentrations lower than 1 μM, thus excluding a role in dioxygen storage or transport.^1^ Consequently, a protective effect of Ngb upon ischemic injuries has been proposed in the view of behavioral studies carried out on mice, yet its biochemical mechanism remains a matter of debate, concerning whether Ngb would perform radical detoxification and/or signal transduction in the activation of antiapoptotic and antioxidant pathways.^2−7^

Although myoglobin (Mb), one of the most studied heme proteins in the scientific literature, and neuroglobin share the typical globin fold composed of eight helices numbered from A to H, the two proteins display only 20% sequence identity. At difference with Mb, in the absence of exogenous ligands, Ngb is endowed with heme iron hexacoordination by two conserved histidines: the distal His64 and the proximal His96.^8−11^ This peculiarity of Ngb induces a complex binding kinetics and conformational rearrangement upon ligation.^12−15^ Ligand binding to the ferrous heme of Ngb is limited by the rate of rupture of the iron–His64 bond, allowing diatomic gases to compete for the vacant sixth coordination position. Notably, the dissociation of His64 and the subsequent diatomic gas (O_2_, NO, CO) ligation to the ferrous iron triggers the sliding of the heme deeper inside the internal Ngb hydrophobic cavity and the movement of loops such as the CDloop and the EFloop. Another remarkable feature, so far only described in murine Ngb, is the almost random heme insertion: the heme has been observed in reversed and canonical orientations compared to the Mb structure, in a 70:30 proportion, due to the loose heme contacts necessary to allow heme sliding.^9,16−19^

Although prior structural studies mainly focused on hampering or facilitating the heme sliding to regulate ligand binding in murine Ngb,^15,17,20^ recent investigations aimed at defining the role of the CDloop in controlling ligand binding kinetics and protein dynamics.^17,18,21^ More in detail, Boron et al. showed the importance of the CDloop sequence and conformation in driving the ferrous iron coordination state by swapping CDloop between Mb and Ngb, leading to the partial loss of hexacoordination in Ngb upon Mb CDloop grafting and *vice versa*.^21^ Moreover, molecular dynamics predictions suggested that Ngb residues, in particular, those forming the CDloop–D-helix segment, interact with cytochrome c and Gα(i) proteins, underlying the molecular basis of Ngb signaling role.^22,23^

Moreover, we reported the effects of an increased CDloop mobility (Gly-loop mutants) on Ngb structure and binding kinetics, demonstrating that the CDloop may act as a distal control for ligand binding.^17,18^ In view of these observations and considering the structure of the Gly-loop mutant (Supporting Information Figure S1), we designed a CDless variant, in which both the CDloop and the D-helix were removed. In this study, we structurally and spectroscopically characterized the CDless mutant, aiming at defining the role of these structural elements in the Ngb structure and ligand binding.

## Results and Discussion

### Crystal Structure of Ferric Ngb CDless

For clarity, we maintained Ngb wild-type residue numbering when referring to Ngb CDless amino acid sequence, in spite of the 13-residue deletion.

**Figure 1:**
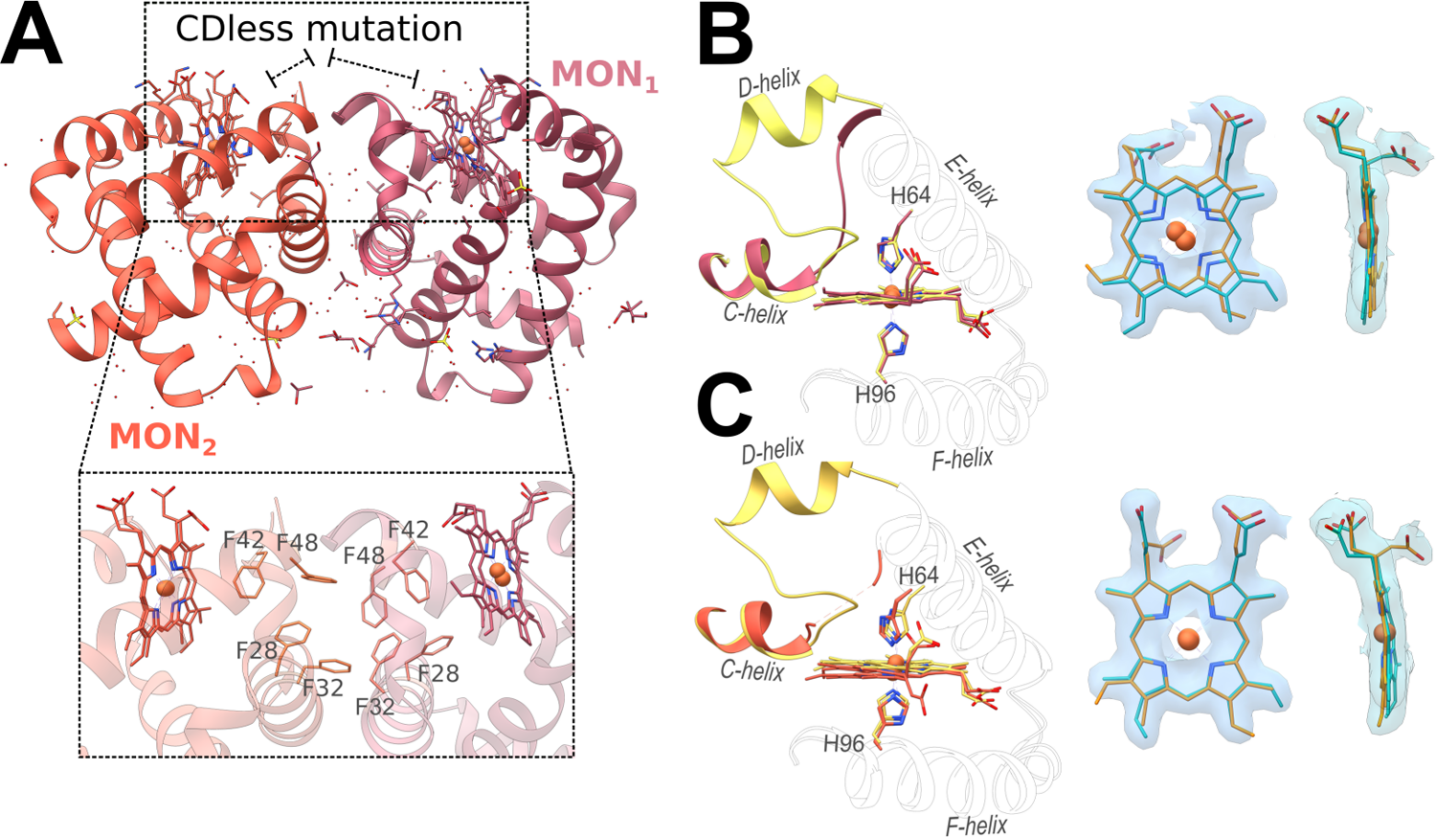
Crystal structure of the neuroglobin CDless mutant determined at a 1.80 Å resolution. (A) The structure of the CDless mutant revealed the presence of two monomers in the asymmetric unit: MON_1_ in dark pink and MON_2_ in dark orange. The interactions between hydrophobic patches of symmetry-related MON_1_ and MON_2_ are shown in the inset. The CElink (protein segment between the C- and E-helices) conformation of Ngb CDless MON_1_ (dark pink in B) and MON_2_ (dark orange in C) is shown in comparison to the CDloop in Ngb wild type (yellow in panels B and C). The corresponding heme insertion positions for MON_1_ (B) and MON_2_ (C) are represented in orange for the canonical insertion and in blue for the reversed one. 2Fo–Fc maps are shown in light blue and contoured at 1σ.

We determined the crystal structure of the ferric murine Ngb CDless mutant at a 1.8 Å resolution, which according to Arcovito et al. is structurally indistinguishable from the reduced ferrous structure.^24^ The analysis of the crystallographic data yielded the observation of two murine Ngb mutant conformational substates (MON_1_ and MON_2_; Figure 1A) in the same crystal. Previously reported structures showed the presence of two heme insertions within the single protein copy found in the asymmetric unit.^9,17,18^ Consistently, the refinement of the heme density for Ngb CDless MON_1_ and MON_2_ indicated the presence of a double heme insertion (Figure 1B,C), along with double conformations for His64 and His96, in proportions similar to those previously reported.^9,17,18^ More in detail, the refinement based on the residual Fc–Fo difference map allowed us to estimate that canonical and reversed hemes are present in a 29:71 and 33:67 ratio in MON_1_ and MON_2_, respectively. The double heme insertion in both monomers leads to somewhat different His64–Fe–His96 geometries (Supporting Information Table S2) as compared to the wild-type protein that may contribute to the heterogeneous reactivity toward CO observed by rapid mixing and flash photolysis reported in the next sections.

As shown in Figure 1B,C, in MON_1_, the positions of the two heme insertions are laterally displaced with respect to each other (Fe–Fe distance = 0.8 Å), while in MON_2_, they are better overlapped, showing a slight rotation of the canonical heme along the β–γ meso axis (Figure 1C), which results in superposed iron atoms, but yields a 0.8 Å distance between the vinyl in C4 (reversed heme) and the methyl in C1 (canonical heme).

Iterative rounds of modeling and refinement enabled us to reconstruct the protein segment between the C- and E-helices (CElink) of MON_1_. However, the poorly defined electronic density for this region in MON_2_ prevented the determination of the CElink structure between Phe42 and Pro59, suggesting a highly mobile conformation (Figure 1B,C). In both monomers, residues belonging to the CElink are scarcely involved in crystal contacts, indicating that their conformations are not influenced by lattice constraints.

In Ngb, the D-helix is stacked above part of the hydrophobic core, namely, Phe28, Phe31, Phe42, Phe49, and Phe61, involved directly (Phe42) and indirectly in heme packing through π–π stacking.^25^ Therefore, the absence of this short helix exposes a hydrophobic patch to the solvent in solution, whereas in the crystal, these surfaces form contacts between the symmetrically related MON_1_ and MON_2_ (Figure 1A, inset). It is worth noticing that in solution Ngb CDless retains its monomeric state (Supporting Information Figure S3)

**Figure 2:**
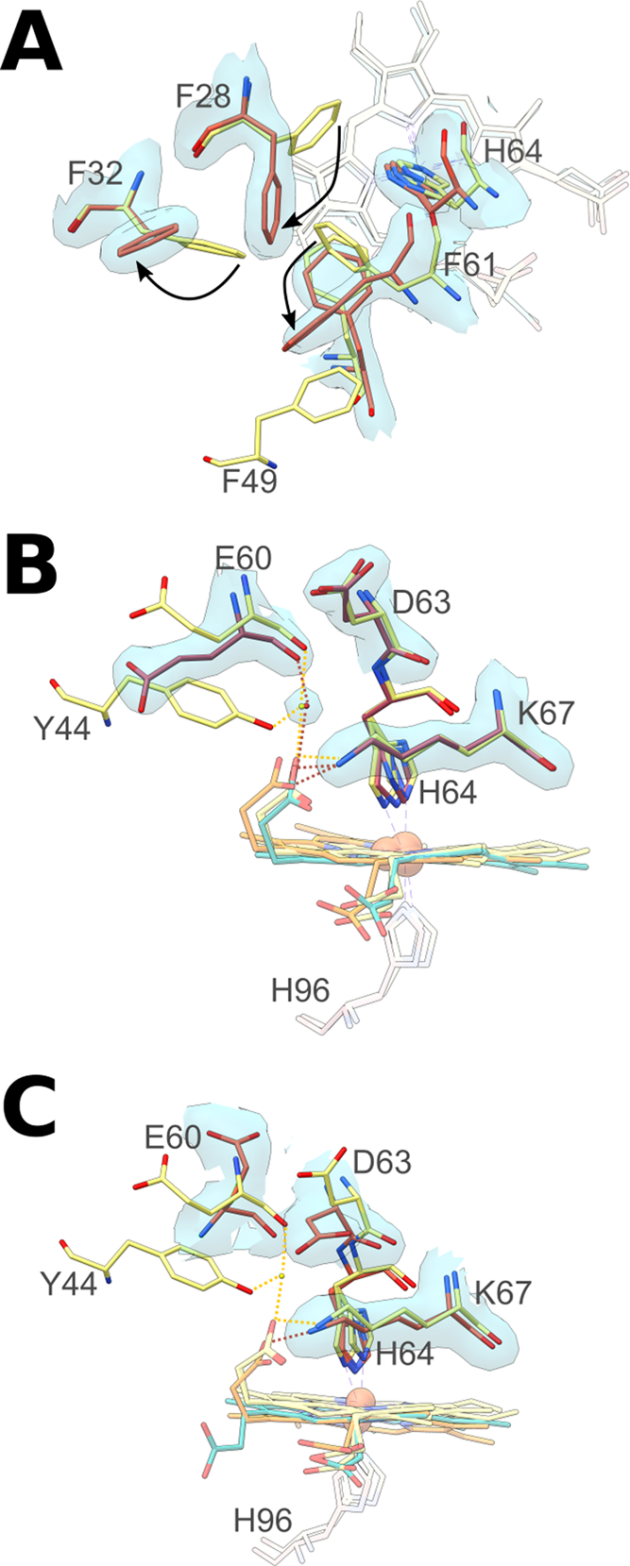
Heme environment in Ngb CDless. Superposition of the structure of Ngb wild type (yellow) on the structure of CDless MON_2_ (A) shows the effects of the CDless mutation on the phenylalanine hydrophobic core of murine Ngb. Panels (B) and (C) show the loss of interactions between the heme propionates and the original CDloop upon the CDless mutation, respectively, in MON_1_ and MON_2_. The wild-type protein (pdb code 1Q1F^17^), CDless MON_1_, and MON_2_ mutants are represented in yellow, pink, and orange, respectively. Canonical and reversed heme insertions are displayed in orange and blue. Water molecules belonging to the wild type and to the mutant structures are displayed as yellow and red spheres, respectively. Dashed lines correspond to H-bonds and electrostatic interactions between distal residues and the heme propionates. Red and yellow indicate Ngb CDless and wild type, respectively. Ngb CDless 2Fo–Fc maps are contoured at 1σ.

Analysis of the structure showed that the lack of D-helix and of Phe49 in Ngb CDless triggers the flipping of Phe61 to the surface of MON_2_, followed by Phe28 to maintain π–π stacking, causing a displacement of the Phe32 side chain toward the bulk (Figure 2A). Conversely, MON_1_, with a defined position of the CElink, appears to preserve the arrangement of the hydrophobic core, retaining the heme crevice observed in Ngb wild type. However, the significant heme displacement observed in MON_1_ (Figure 1B,C) may be attributed to a lifting of Phe42 (not shown), which is absent in MON_2_.

For both heme insertions of MON_1_ and for the canonical insertion of MON_2_, the propionates assume the same position as in Ngb wild type, whereas, for the reverse heme insertion of MON_2_, the C7 propionyl group adopts a proximal orientation owing to the loss of interaction with Lys67 and Tyr44 (Figures 1C and 2B,C). In Ngb wild type, the interaction between the distal propionate with Lys67 and Tyr44 through a water molecule anchors the CDloop to the heme, forming a barrier that affects His64 dissociation from the heme, which is a requisite for ligand binding.^17,26−29^

### Resonance Raman (RR) Spectroscopy

**Figure 3:**
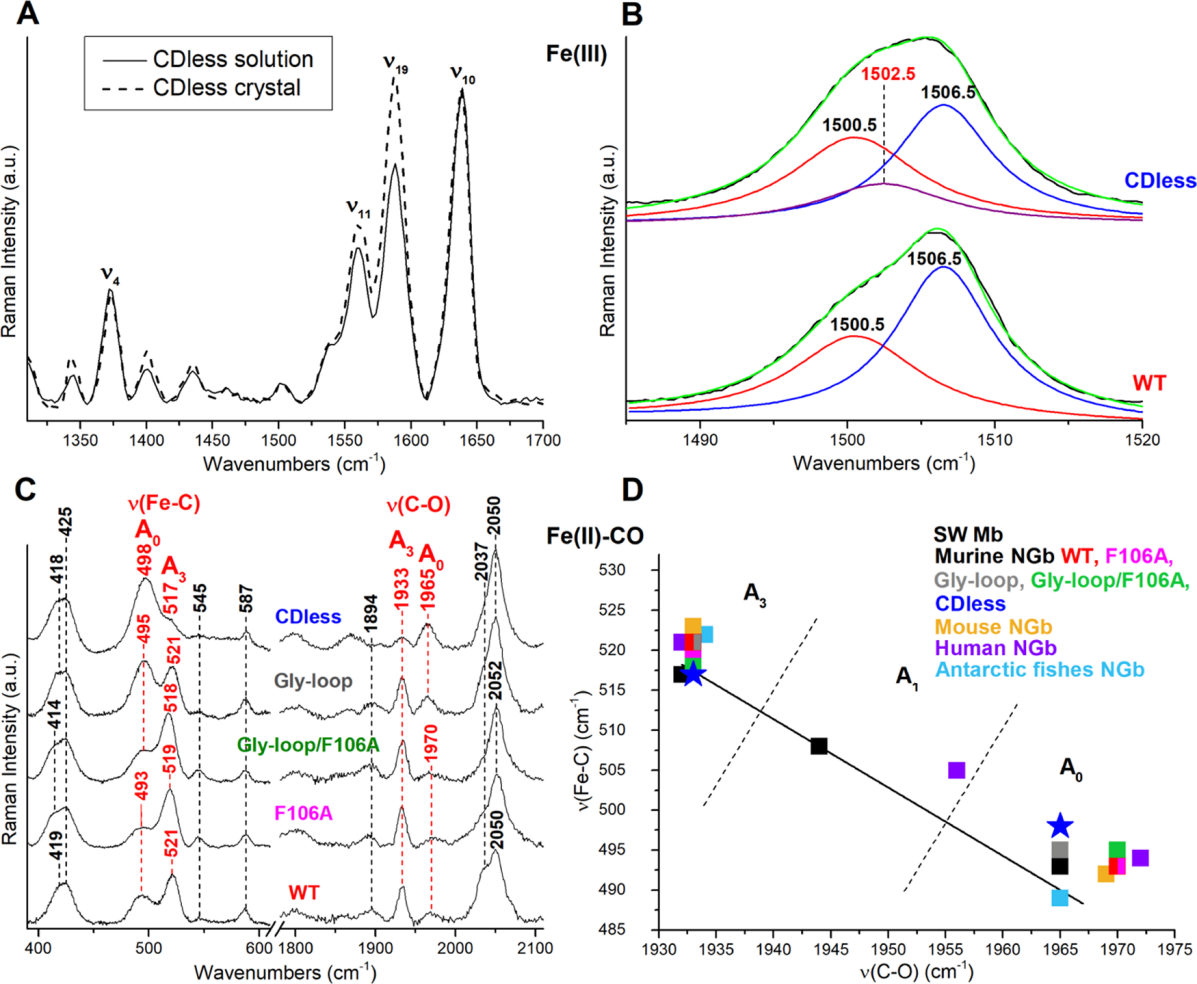
Effect of the CDless mutation on the heme environment probed by resonance Raman spectroscopy. (A) Comparison of the high-frequency RR spectra of the CDless mutant in solution (black line) and in crystal (dotted line) states. The spectra have been normalized to the ν_10_ band at 1639 cm^–1^. Experimental conditions: λ_exc_ 514.5 nm; solution: laser power at the sample 2 mW, average of six spectra with a 30 min integration time; crystal: laser power at the sample 5 μW, average of 26 spectra with a 260 min integration time. (B) Curve-fitting analysis of the v_3_ region of the ferric wild type (bottom) and CDless mutant (top) obtained with the 413.1 nm excitation wavelength (for the experimental conditions, see Supporting Information Figure S4). The bandwidths are 10.5 cm^–1^ for the bands at 1500.5 and 1502.5 and 8.5 cm^–1^ for the band at 1506.5 cm^–1^. (C) RR spectra in the low- (left) and high- (right) frequency regions of the Fe(II)–^12^CO complexes of the CDless mutant (this work), and the WT, F106A, Gly-loop/F106A, and the Gly-loop mutant Ngbs.^18^ The frequencies of the ν(Fe–C) and ν(C–O) stretching modes are labeled in red (open (A_0_) and closed (A_3_) forms). The spectra in panels (B) and (C) have been shifted along the ordinate axis for visualization. Experimental conditions: λ_exc_ 413.1 nm; CDless mutant: laser power at the sample 550 μW, average of 18–27 spectra with a 180–270 min integration time for the low- and high-frequency regions. Wild type (WT), F106A, Gly-loop/F106A, and the Gly-loop mutants (see ref (18) for details). (D) Back-bonding correlation line (black) of the ν(Fe–C) and ν(C–O) stretching frequencies of various Ngbs,^17,30−33^ as reported in Supporting Information Table S3, together with the corresponding data of sperm whale Mb (swMb). The dotted lines indicate the approximate delineation between the frequency zones of the A_0_, A_1_, and A_3_ forms. The humans Ngb and swMb show also a third weak H-bonded conformer (A_1_) at 505/1956 and 508/1946 cm^–1^, respectively.

RR spectra in solution and for the crystal of ferric Ngb are very similar, indicating that the conformational substates and heme structural heterogeneity identified by crystallography are not artifacts due to crystal packing (Figure 3A).

The comparison of the solution RR spectra of ferric CDless and wild-type proteins shows that this mutant occurs as a pure low-spin hexacoordinate species (6cLS) like Ngb wild type, confirming that the loss of the CDloop and the D-helix does not affect bis-histidyl hexacoordination (Supporting Information Figure S4). In agreement with the crystal structure, the overall heme orientation is unaltered since RR spectra reveal the presence of two 6cLS species with different core sizes, assigned to the planar reversed conformer (characterized by ν_3_, ν_2_, and ν_10_ bands at 1500, 1576, and 1637 cm^–1^, respectively) and a distorted canonical conformer (ν_3_, ν_2_, and ν_10_ core size bands at 1506, 1578, and 1639 cm^–1^, respectively). Moreover, we observe two vinyl stretching modes (1620 and 1631 cm^–1^) and three vinyl bending modes (404, 416, and 428 cm^–1^) in Ngb CDless, arising from the overlap of the signal from vinyl groups in *cis* and *trans* conformations, as observed for the wild-type protein.^18^ The lack of frequency shift between these modes for the native protein and the mutant indicates that the interactions between the vinyl and neighboring residues are not affected by the CDless mutation.

As deduced from the relative intensities of the Raman core size marker bands, the amount of the canonical heme insertion increases in the CDless mutant at the expense of the reversed insertion with respect to the wild type. The increase of the canonical heme insertion with respect to the reversed form tends to be a common feature in CDloop-altered mutants (Supporting Information Figure S4).^17−19^ RR does not give any clear evidence of the presence of two protein conformations corresponding to MON_1_ and MON_2_ found in the crystal. However, in the 1450–1550 cm^–1^ region, where only the ν_3_ core size marker band is expected, the solution spectrum of the CDless mutant cannot be fitted like it has been done for Ngb wild-type and other mutants (two bands at 1500.5 and 1506.5 cm^–1^, Supporting Information Figure S5). A more precise result can be obtained if a third ν_3_ band at 1502.5 cm^–1^ is considered. This finding suggests the presence of at least another form, in line with the results of diffraction experiments, supporting the presence of two substates each containing a reverse and a canonical heme insertion (Figure 3B). In the ν_2_ and ν_10_ vinyl stretching mode regions, many bands overlap (Supporting Information Figure S6), preventing the resolution of additional heme conformers. The complete assignment of the core size marker bands obtained upon Soret (λ_exc_ at 413.1 nm) and Q-bands (λ_exc_ at 532 nm) excitations in both depolarized and polarized (data not shown) lights is reported in Supporting Information Table S3.

Ferrous Ngb CDless and Ngb wild type showed similar 6cLS RR spectra (data not shown), as reported for other mutants,^17^ and small changes were only observed in the ν_10_/vinyl stretching region. However, the RR spectra of the Fe(II)–CO complexes of Ngb CDless are markedly different from the wild type and other mutants (Figure 3C). Consistently with previous results, the back-bonding correlation between the ν(Fe–C) and ν(C–O) stretching mode frequencies (Figure 3D) compared with various Ngbs^17,30−33^ and sperm whale Mb^34^ listed in Supporting Information Table S4 indicated the existence of two protein conformer populations: one (A_1_) with a closed cavity, whose CO is H-bonded to His64 (ν(Fe–C) = 517 cm^–1^ and ν(CO) = 1933 cm^–1^), and a remarkably abundant population (A_0_) endowed with an open cavity (ν(Fe–C) = 498 cm^–1^ and ν(CO) = 1965 cm^–1^). The assignment of the ν(Fe–C) and ν(C–O) stretching modes is obtained by isotopic labeling with ^12^CO and ^13^CO (Supporting Information Figure S7).

### Rapid Mixing at 25 °C

The wild-type binding kinetics for CO shows a monoexponential behavior in agreement with published results (Supporting Information Figure S8A),^17,20^ while binding of CO to Ngb CDless is best described by a biexponential relaxation (Supporting Information Figure S8B), as reported for other murine Ngb mutants, such as F106A, Gly-loop/F106A, and Y44D.^17,35^ Commonly, a biphasic behavior is attributed to the presence of two Ngb populations and indeed the relative amplitudes determined for CO binding to Ngb CDless (A__slow_ = 27% against A__fast_ = 73%) are reminiscent of the relative 30:70 heme insertions observed in Ngb wild type and mutants.^9,16−18^ The Ngb CDless structure shows canonical and reversed hemes in a 29:71 and 33:67 ratio in MON_1_ and MON_2_, respectively, and we propose to assign the fast phase (A__fast_ = 73%) to the Ngb CDless population endowed with the reversed heme insertion and the slow phase (A__slow_ = 27%) to the population with the canonical one. It is worth mentioning that a double exponential relaxation would be expected also for the wild-type protein, where the double heme insertion is also present. However, unlike for the CDless mutant, the two phases are not distinguishable for the wild-type protein, most likely owing to similar rate constants. Admittedly, the fitting of CO binding traces of Ngb wild type with a single exponential decay is less than optimal but in agreement with previous results, adding a second decay yields unstable results.

**Figure 4:**
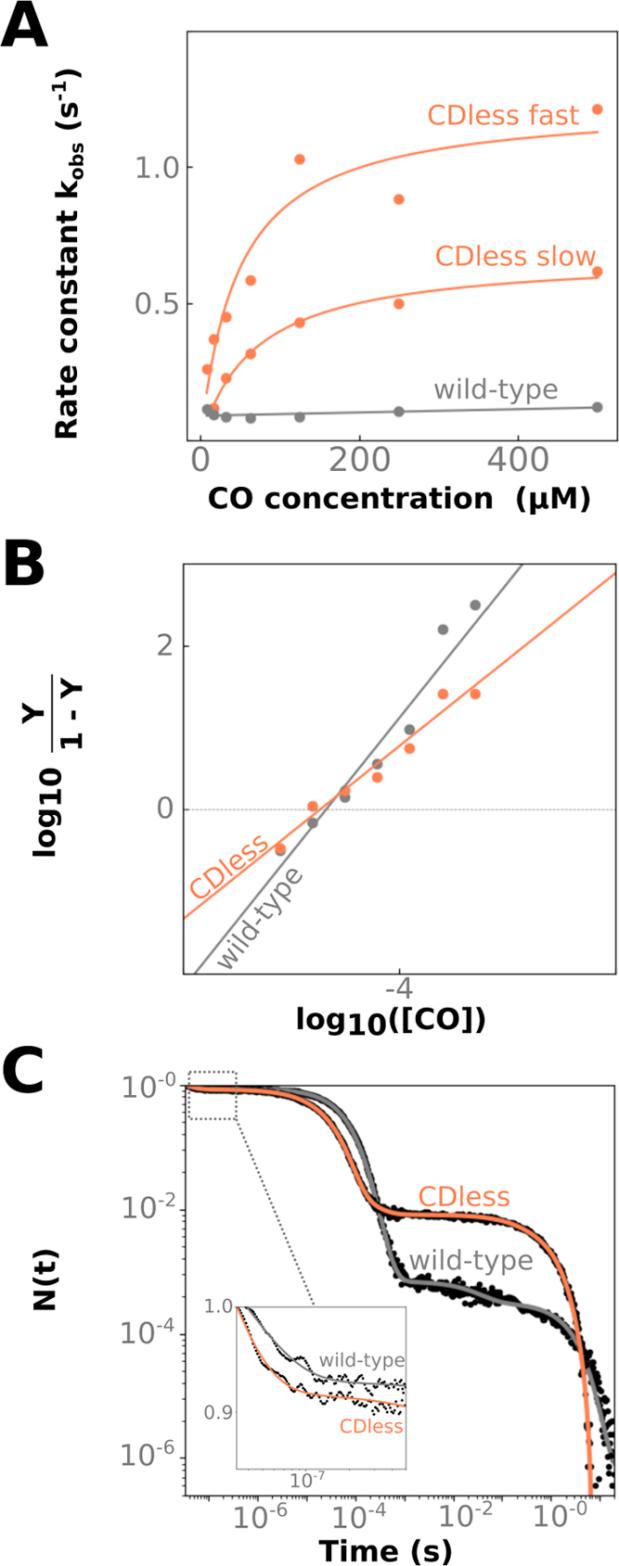
CO (re)binding kinetics of neuroglobin wild type (gray) and CDless (orange) at 25 °C. (A) Rapid mixing kinetic traces for Ngb wild type and CDless were fitted as mono- or biexponentials and corresponding rate constants *k*_obs_. The dependence of rate constants on CO concentration is fitted (full lines) according to Hargrove and collaborators;^14^ the values are reported in Supporting Information Table S7. (B) The fraction Y of Ngb bound to CO was extracted from the overall amplitude of the kinetic traces, and log 10(*Y*/1-*Y*) was plotted as a function of the CO concentration. Data were linearly fitted to determine the overall CO affinity c_50_. (C) CO rebinding kinetics was followed by laser flash photolysis at 25 °C at 0.2 atm CO. Data are reported as the progress curve representing the fraction of deoxy molecules, N(t), as a function of time after photolysis. Fitting curves using exponential decay functions are superimposed to the experimental data (black dots) and colored, respectively, in gray and orange for Ngb wild type and CDless. The inset is a close-up view of the kinetic trace at an early time range.

Moreover, Ngb CDless shows increased velocities upon ligand binding (*k*_obs_slow_ = 0.62 ± 6.7 × 10^–3^ s^–1^ and *k*_obs_fast_ = 1.2 ± 2.1 × 10^–2^ s^–1^ at 500 μM CO; Figure 4A) with respect to Ngb wild type (*k*_obs_ = 0.13 ± 1.4 × 10^–3^ s^–1^ at 500 μM CO), but it is less affected than other CDloop-altered mutants endowed with increased structural flexibility.^17^ The proximal position of the C7-propionate (reversed heme) is consistent with enhanced CO binding kinetics, owing to a facilitated His64 dissociation from the heme and swinging out of the pocket. Consistently, a previously described Gly-loop mutant endowed with significantly faster CO binding kinetics showed destabilization of the heme propionate that blocks the pocket external access, for both canonical and reversed heme insertion, and provided structural evidence of swung-out His64 in Ngb.^17^

Kinetics investigations performed on murine Ngb demonstrated that ligand binding occurs according to a two-step mechanism: the first, rate-limiting event is the rupture of the His64–Fe bond, while the second step consists of fast CO ligation to the iron sixth coordination position.^26−28,36^ The latter event is endowed with a higher velocity at high CO concentrations. Therefore, at high enough CO concentrations, the observed binding rate constant *k*_obs_ determined by rapid mixing approximates the His64 dissociation rate *k*_off_. Interestingly, the affinity for CO of the CDless and wild-type variant is identical within experimental error (c_50_(CDless) = 19.0 ± 0.8 μM, while c_50_(wild-type) = 20.6 ± 1.1 μM; Figure 4B); therefore, we hypothesized that the apparent rate constant for CO dissociation (*k*_off_) is increased in the CDless mutant.

### Laser Flash Photolysis at 25 °C

To gain further insight into the role of the CDloop and D-helix in tuning the balance between heme binding to the endogenous His64 and to exogenous diatomic ligands, we followed the CO rebinding kinetics of Ngb CDless after nanosecond laser flash photolysis (LFP).

The CO (0.2 mM) rebinding kinetics for Ngb wild type and the CDless mutant (Figure 4C) shows that the overall curves are similar and composed of three well-defined kinetic phases, in agreement with that described for murine^27^ and human^26^ Ngb. A geminate rebinding phase in the ns−μs time scale, corresponding to CO rebinding from within the protein matrix, is followed by a CO concentration-dependent phase on the long microsecond time scale that can be assigned to the competition between the bimolecular rebinding of CO and the association of endogenous His64. A slower process is observed in the seconds time range, which we attribute to the disappearance of the transient bis-histidyl species as CO rebinds from the bulk (Supporting Information Figure S9). LFP traces were fitted with a sum of exponential decay functions, and the extracted lifetimes are reported in Supporting Information Table S5.

Figure 4C (inset) shows that the amplitude of Ngb CDless geminate rebinding (16%) increases to about 30% with respect to Ngb wild type (12%), the latter one being in agreement with that previously observed.^27,28,37^ Moreover, the rate of the geminate rebinding shows a twofold increase (5.4 × 10^7^ s^–1^ vs 2.9 × 10^7^ s^–1^ for the wild type). The possible involvement of nearby temporary docking sites is suggested by the presence of a process of small amplitude endowed with a lifetime of ∼7 μs that can be identified only in the CDless mutant (Supporting Information Table S5) and that may arise from the migration of photodissociated CO molecules to kinetic traps, as observed for murine and icefish Ngbs at low temperature.^27,31,38^ We used a simple model (eq 1) to describe the geminate rebinding on the nanosecond time scale when His64 recombination is negligible, where we assume that the photodissociated ligand may either exit the protein matrix or rebind from the distal pocket
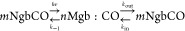
1

Using the amplitude (Φ) and the apparent rate constant for the geminate rebinding (*k*_gem_), this reaction model allows us to estimate the exit rate constant *k*_out_*= (*1*–*Φ*)* × *k*_gem_ and the rebinding rate *k*_*–*1_ = Φ *k*_gem._(39)

The rebinding rate *k*_*–1*_ shows a nearly threefold increase for the mutant (3.5 × 10^6^ s^–1^ for the wild-type protein vs 8.7 × 10^6^ s^–1^ for the mutant). Interestingly, *k*_out_ is almost twofold higher for Ngb CDless (4.6 × 10^7^ s^–1^) than for the wild-type protein (2.6 × 10^7^ s^–1^), suggesting that the CDless mutation favors a CO escape route located between the distal heme pocket and the solvent. The elimination of the CDloop–D-helix unit, on top of the distal heme cavity, might account for the enhanced escape of photodissociated CO and is consistent with the observation by RR of a predominant open conformation in the CO-bound CDless mutant.

The dominant process observed in Figure 4C accounts for the bimolecular CO rebinding from the solvent, where the apparent rate for Ngb CDless is higher than that of the wild-type protein and compatible with the increased amplitude determined for the geminate phase. Interestingly, the second-order reaction between CO and the pentacoordinated species is faster for the mutant, but, in both proteins, the bimolecular process is described by two exponential phases, although with different proportions. The relative amplitudes of the second-order CO binding for Ngb wild type (65/35%) seem to account for the double heme insertion (70/30%^9^), whereas in the CDless, the faster event becomes absolutely dominant (93%) on the slower one (7%). This different behavior most likely reflects a structural relaxation upon endogenous ligand binding. A competition between CO rebinding and His64 recombination for the heme iron takes place during the bimolecular phase. The formation of the His64 internally hexacoordinated population is more efficient for the mutant (Figure 4C), indicating that the CDless mutation substantially increases the His64 binding rate, as also deduced from rapid mixing experiments (Supporting Information Table S6). The more efficient accumulation of hexacoordinated species is likely the basis of the observed higher photolability of the CO complex for the CDless mutant in resonance Raman experiments.

The final kinetic event, extending to several seconds, reflects His64 dissociation from the heme and restoration of the more stable bond with CO; LFP results suggest that the CDless mutation leads to an increase in His64 dissociation velocity. This is in agreement with rapid mixing experiments (Figure 4A,C) from which we estimated His64 binding (*k*_H_) and dissociation (*k*_–H_) rates (*k*_–H_(LFP) = 0.52 s^–1^ vs *k*_obs_(rapid mixing) = 0.62 s^–1^ (Supporting Information Table S6).

However, we note that in LFP experiments, given the small amplitude of the process, it was not possible to identify reliably two distinct steps in the decay of the hexacoordinated species. These discrepancies on the long time scales could also arise from the fact that rebinding of photodissociated CO occurs rapidly, preventing full relaxation of the protein on the millisecond time scale. Indeed, in LFP, CO molecules are rapidly photodissociated and they rebind to a partially relaxed protein conformation that may fully or partially preserve the structural and functional properties of the pentacoordinated carboxy-species, whereas in rapid mixing experiments, the CO molecule encounters Ngb in a fully relaxed hexacoordinated form and binds the heme after displacement of His64.

Finally, in the CO concentration range used in the rapid mixing experiments, the CO binding to Ngb wild type can be described by a single exponential decay, where the rate constant is virtually independent of CO concentration. Since this process represents the rate-limiting step for the overall formation of the CO complex, the influence of the CDloop–D-helix unit on the bis-histidyl hexacoordination appears to play a key role in tuning the reactivity of the heme.

### MD Simulations

Molecular dynamics (MD) simulations of Ngb CDless in a crystal environment were performed in a single unit cell, comprising 12 protein replicas (six replicas of MON_1_ and six of MON_2_). One-third of the replicas were randomly chosen to be simulated with the heme in its canonical insertion.

**Figure 5:**
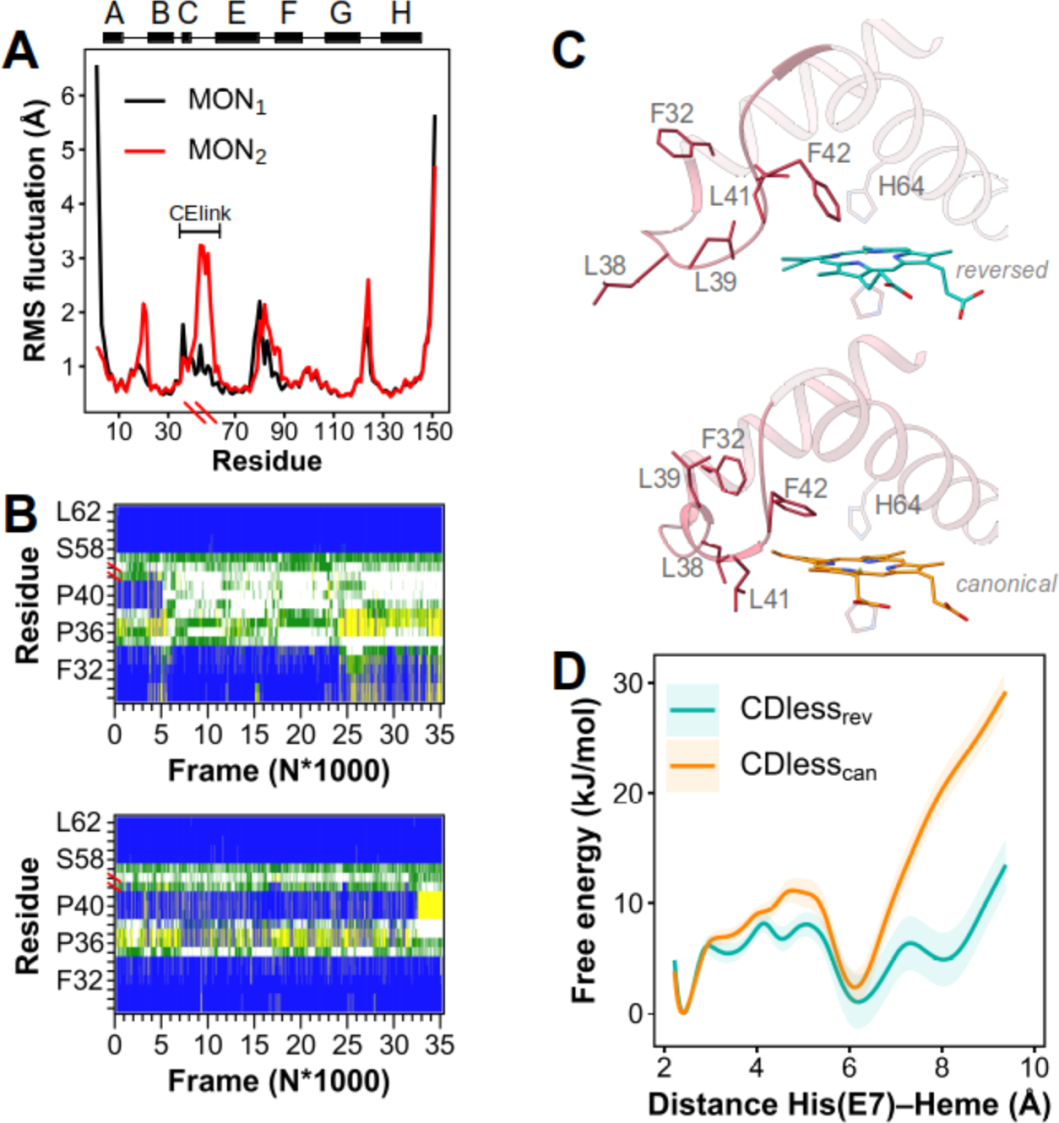
Molecular dynamics simulations of Ngb CDless. (A) Root-mean-square fluctuations of Cα atoms from the cumulative simulations of MON1 and MON2 in crystallo. Secondary structures and helices numbering are represented in black on top of the graph. (B) Influence of the heme insertion mode on the C-helix stability in Ngb CDless in solution. Top panel: secondary structure adopted by the C-helix-CElink in the presence of the reversed heme insertion. Bottom panel: in the presence of the canonical one. Helices are represented in blue, coils are in white, turns are in yellow, and bends are in green. (C) Close-up view of the C-helix-CElink in the presence of either the reversed (top) or the canonical heme insertion (bottom). Porphyrin groups are displayed in blue (reversed heme insertion) or orange (canonical heme insertion). (D) Free energy associated with the His64 displacement in Ngb CDless embedding either the reversed or the canonical heme.

Figure 5A describes the root-mean-square fluctuation (RMSF) of Cα atoms determined from the cumulative simulations of MON_1_ and MON_2_*in crystallo*. The main differences in RMSF between MON_1_ and MON_2_ are observed for the CElink, where MON_2_ displays a significantly higher structural disorder and, to a lesser extent, at the level of the AB corner.

The cluster analysis performed with a 1 Å cutoff on the cumulative trajectories of MON_1_ and MON_2_ revealed that a single cluster of MON_1_ contained up to ∼76% of the total conformations adopted by MON_1_, which suggests a rather conserved conformation within the simulated crystal lattice (Supporting Information Figure S10), whereas the 10 most populated clusters obtained for MON_2_ grouped no more than ∼72% of the total simulated structures, highlighting a certain degree of conformational freedom in the crystal state for this monomer (Supporting Information Figure S11). The most representative structures, reported in Supporting Information Figures S10 and S11 for MON_1_ and MON_2_, are in agreement with the crystallographic structures. Notably, the CElink in MON_2_ adopts a plethora of conformations, which suggests a higher loop mobility, consistent with its poorly defined electronic density.

Supporting Information Figure S12 reports the secondary structure assignment for residues Ala29–Leu49 in every replica simulated in the crystal unit cell. In this environment, the C-helix (residues Pro36–Leu41) is quite stable independently from the heme insertion (Supporting Information Figure S12). Notably, simulations *in crystallo* showed that the first turn of the E-helix in MON_2_ resulted in being less stable than in MON_1_.

Simulations performed on Ngb CDless in solution revealed that the heme insertion affects the conformation of the C-helix, which appears structured if the protein contains the canonical insertion but completely unstructured in the presence of the reversed insertion. When Ngb CDless embeds the heme in canonical insertion, the C-helix appears stable (Figure 5B,C), with Leu38 forming sticky hydrophobic contacts with one of the heme vinyl groups. However, with the reversed insertion, the contacts between Leu38 and the heme methyl group are less favorable, resulting in an unstable C-helix. The ensuing unwinding of the C-helix yields the displacement of the Phe42 side chain no longer parallel to the heme plane, while Leu38 and Phe32 side chains point toward the solvent. Consequently, the heme methyl and vinyl groups are packed against the Leu39 side chain for the reverse heme conformation.

We also simulated the Ngb CDless in the CO-bound form to obtain information on the dynamics of His64. The heme insertion mode also affects the displacement of His64, in an N_δ_ tautomeric form, immediately after the rupture of the heme iron–His64 bond. Two representative structures of carboxy Ngb CDless obtained from MD simulations are reported in Supporting Information Figure S13A. His64 swings outside in the presence of a reversed heme insertion. In the structure with the canonical insertion, however, His64 peculiarly flips inside the distal pocket (Supporting Information Figure S13B). Such a “swing in” displacement of His64 is coupled with the unfolding of the first two turns of the G-helix (residues Arg89–Ser92).

The two distinct conformations of the C-helix (*i.e*., structured and unstructured) confer a different degree of flexibility to Ngb CDless, and a structured and rigid C-helix might constitute a barrier to His64 swinging out from the heme pocket (Supporting Information Figure S13B). In agreement with this consideration, we found that the C-helix conformation, and, therefore, the heme insertion, strongly influences the free energy of the His64 displacement (Figure 5D). Namely, for the canonical insertion, the high free-energy penalty, associated with the His64 displacement, suggests that this heme insertion favors the rebinding of His64, whereas the low free-energy barrier corresponding to the His64 displacement in the presence of the reversed heme insertion implies that the binding of an exogenous ligand to the sixth coordination position is favored.

### Final Remarks

We investigated the role of the CDloop and D-helix in neuroglobin (Ngb), which are the structural elements most likely involved in allowing Ngb to carry out a signaling function, using the CDless mutant where these structural elements are absent.

The X-ray structure and resonance Raman spectra of the ferric form indicate that their excision allows maintenance of heme hexacoordination; moreover, laser flash photolysis and rapid mixing experiments showed moderately increased His64 k_-H_ and k_H_ but unaffected ligand affinity. We therefore observed that upon elimination of the CDloop–D-helix module Ngb maintains a somewhat rigid protein core, as reflected by functional parameters. At difference, it was reported that introducing flexibility in the CDloop, as in the Gly-loop mutant series, markedly increased ligand binding velocity and affinity.^17^ This is consistent with the scenario in which the CDloop–D-helix unit represents a structural feature that couples ligand binding to a structural transition, compatible with a signaling role.

Additionally, the absence of the CDloop that contacts one of the heme propionates via Tyr44 may lower the barrier against distal His64 swinging out as indicated by resonance Raman spectroscopy of the CO-bound form, which has a predominantly open heme cavity. MD simulations confirm an enhanced tendency of His64 to swing out for the reversed heme conformer. Since the fraction of Ngb CDless molecules endowed with reversed heme insertion is about 70%, it is tempting to assign to this population the 70% fast phase observed in CO rapid mixing experiments. Finally, laser flash photolysis experiments show that the ligand exit rate constant is almost twofold higher for Ngb CDless than for the wild type. This may be ascribed (i) to the lack of the CDloop–D-helix module and the consequent formation of a preferential escape route that was marginally observed in wild type^40^ and (ii) to the enhanced tendency of His64 to swing out for the reversed heme insertion, which could provide a direct escape route for the photolyzed CO.

Data reported by Boron et al.,^21^ who swapped the CDloop sequences between Ngb and sperm whale Mb to address its role in determining heme coordination state, led to the conclusion that this region affects hexacoordination dynamics, but it is not the main structural determinant of this remarkable Ngb feature. This is in line with our results, since we observed the retention of hexacoordination upon deletion of the CDloop–D-helix albeit with the variations in kinetics summarized above.

In conclusion, upon excising the CDloop–D-helix structural unit, Ngb retains rigidity and reduces histidine gating control via structural rearrangement of the CDloop–D-helix. We hypothesized that the CDless mutation may abolish signaling, and such a mutant may be used as a tool to probe the physiological roles of murine neuroglobin *in cellulo* or *in vivo*. This could be confirmed only by experiments in cells or *in vivo* in which we tentatively predict that the CDless mutant, only marginally affected from the functional point of view, should be unable to trigger neuroprotection upon hypoxic/oxidative insults if the CDloop–D-helix unit acts as a triggering module. Conversely, in the context of the radical scavenging hypothesis, the CDless mutant might retain Ngb wild-type protective effect, at least to some extent.

## Methods

### Expression and Purification

The gene corresponding to murine Ngb CDless (Figures S1 and S2) was synthesized and cloned in pET14b. Wild type and Ngb CDless were expressed and purified as described previously.^24^

### Rapid Mixing

Reduced Ngbs (8.4 μM for the wild type and 4.0 μM for the CDless mutant) were anaerobically and symmetrically mixed with CO-equilibrated buffer solutions. Experiments were performed in 100 mM HEPES pH 7.4 at 25 °C and followed at 426 nm using a 1 cm light path Applied Photophysics stopped-flow instrument. Three to four kinetic traces were collected and averaged per CO concentration.

### Laser Flash Photolysis

The laser flash photolysis setup has been described elsewhere.^41^ Absorbance changes upon CO photodissociation were monitored at 436 nm using a monochromatized cw output of a 150 W Xe arc lamp. The sample holder was temperature-controlled with a Peltier element, allowing temperature stability of at least 0.1 °C. Three traces were collected and averaged per time course.

### Crystallization, X-ray Crystallography, and Data Analysis

Ngb CDless crystals were obtained using the hanging drop vapor diffusion method in 0.8 M ammonium sulfate, 100 mM bis–tris pH 6.0, and 3% isopropanol. Data collection was performed at the I04 beamline (DIAMOND, United Kingdom). The structure of the Ngb CDless mutant was solved by molecular replacement (MOLREP, CCP4 7.0.078 release^42^) using 6H6I^17^ as a model. Refinement and model building were carried out using Refmac5 (CCP4 7.0.078 release^43^) or Phenix v1.14–3260^44^ and Coot 0.8.9.2 respectively.^45^ Structure determination statistics are reported in Supporting Information Table S1.

### Resonance Raman Spectroscopy

The resonance Raman (RR) spectra of crystals, mounted in capillaries, were obtained using a Renishaw RM2000 Raman microscope with a 514.5 nm line (Ar^+^ laser). The RR spectra of samples in solution in 100 mM HEPES pH 7.4 were obtained with excitation wavelengths of 514.5 (Ar^+^), 413.1 (Kr^+^), and 532 nm (diode laser Cobolt Samba 300). All of the experimental conditions, including the experiments in polarized light, have been described previously.^17,18^ All RR measurements were repeated several times to ensure reproducibility, and all spectra were baseline-corrected. The CO complexes have been obtained as reported in.^17^

### Molecular Dynamics Simulations

The crystal unit cell was obtained by applying the P63 symmetry transformations to MON_1_ and MON_2_, and two different neuroglobin CDless copies were found in the asymmetric unit. The missing residues were modeled by Molecular Operating Environment.^46^ The unit cell was solvated with ∼8000 TIP3P water molecules^47^ and 48 Na^+^ ions, with two successive solvent additions, each followed by a solvent relaxation session. The system was thermalized at 300 K in 10 ns and equilibrated for 50 ns. A 500 ns simulated tempering^48^ simulation was then performed at a constant volume with the temperature ranging from 300 to 420 K in steps of 5 K. Starting coordinates of Ngb CDless in solution were taken from MON_1_, one of the two monomers found in the asymmetric unit by X-ray crystallography. The protein was placed in a dodecahedron box with ∼9000 TIP3P water molecules^47^ and 4 Na^+^ ions. After a thermalization of 10 ns and an equilibration run of 100 ns, simulated tempering simulations (1 μs) were done at a constant volume and with temperatures ranging from 300 to 420 K in steps of 10 K. Pulling simulations^49^ coupled with simulated tempering^48^ were performed after breaking the bond between the heme and the distal histidine. The distance between heme iron and the distal histidine N_ε_ atom was the reaction coordinate. Representative configurations along the reaction coordinate were extracted, and 37 umbrella sampling simulations were spawned. Free-energy profiles were obtained using the weighted histogram analysis method.^50^ Starting coordinates of carboxy Ngb CDless in solution were generated from representative configurations of Ngb CDless with displaced distal histidine, after CO had been bound to the sixth coordination position. After the same equilibration procedure used for unliganded Ngb CDless, 300 ns simulated tempering simulations^48^ were performed. All of the simulations were performed using the GROMACS 2019.6 software package^51^ and CHARMM36m force field.^52^ The simulation protocols were the same as reported in ref (17).

## Supporting Information Available

This supporting material provides detailed experimental methods, sequence alignments, crystallographic tables, rate constant tables, and additional RR spectra, which aim at a better understanding and interpretation of the results reported in this work (PDF)

## Notes

Atomic coordinates were deposited with PDB accession number 7OHD.
