# Supplementary material for: Probing the Role of Murine Neuroglobin CDloop–D-Helix Unit in CO Ligand Binding and Structural Dynamics
Source: ACS Chem Biol. 2022 Jul 7;17(8):2099–108. doi: 10.1021/acschembio.2c00172 (PMC9396615; doi:10.1021/acschembio.2c00172)
Supplement: Supplementary file 1 — cb2c00172_si_001.pdf [file cb2c00172_si_001.pdf]

Supplementary Materials

Probing the role of murine neuroglobin CDloop-D-helix unit in CO ligand binding and structural dynamics.

Cécile Exertier[1]*, Federico Sebastiani[2]*, Ida Freda[1], Elena Gugole[1], Gabriele Cerutti[3], Giacomo Parisi[4], Linda Celeste Montemiglio[5], Maurizio Becucci[2], Cristiano Viappiani[6], Stefano Bruno[7], Carmelinda Savino[5], Carlotta Zamparelli[1], Massimiliano Anselmi[8]*, Stefania Abbruzzetti[6]*, Giulietta Smulevich[2]* & Beatrice Vallone[1]*

[1]Dipartimento di Scienze Biochimiche "A. Rossi Fanelli", Sapienza, Università di Roma, Piazzale A. Moro 5, I-00185 Rome, Italy

[2]Dipartimento di Chimica "Ugo Schiff", Università di Firenze, Via della Lastruccia 3-13, I-50019 Sesto Fiorentino, Italy.

[3]Zuckerman Mind Brain Behavior Institute, Columbia University, 3227 Broadway, New York, NY 10027, USA.

[4]Center for Life Nanoscience, Istituto Italiano di Tecnologia, Viale Regina Elena, 291, I-00161 Rome, Italy.

[5]Institute of Molecular Biology and Pathology, National Research Council, Piazzale A. Moro 5, 00185, Rome, Italy

[6]Department of Mathematical, Physical and Computer Sciences, University of Parma, Parco Area delle Scienze, 7/A, I-43124 Parma, Italy

[7]Department of Food and Drugs, University of Parma, Parco Area delle Scienze 27/A, I-43124 Parma, Italy

[8]Theoretical Physics and Center for Biophysics, Saarland University, Campus E2 6, 66123 Saarbrücken, Germany

*These authors contributed equally to this work.

[#]To whom correspondence should be addressed: Tel: +390649690276 E-mail: beatrice.vallone@uniroma1.it

Supplementary Figures S1 to S13
Supplementary Tables S1 to S6
References

Supplementary methods

Rapid mixing.

Degassed and reduced solutions were obtained by extensive $N_{2(g)}$ equilibration and addition of a three-fold stoechiometric excess of sodium dithionite.

Reduced Ngb variants (8.4 μM for the wild-type and 4.0 μM for the CDless mutant) were anaerobically and symmetrically mixed with CO-equilibrated buffer solutions. Rapid mixing experiments were performed in 100 mM HEPES pH 7.4 at 25°C and followed at 426 nm using a 1 cm-lightpath Applied Photophysics stopped-flow instrument. Three to four kinetic traces were collected and averaged per CO concentration.

Experimental data were analyzed QtiPlot and figures were made using Matplotlib. Monophasic traces were fitted as $y_0 + A*\exp(-k_{obs}*t)$ while bi-phasic trends were fitted as $y_0 + A_{fast}*\exp(-k_{obs\_fast}*t) + A_{slow}*\exp(-k_{obs\_slow}*t)$ where y_0 is the offset, A, A_{fast} and A_{slow} are the amplitudes and k_{obs}, k_{obs_fast} and k_{obs_slow} are the CO binding rate constants.

Crystallization and X-ray crystallography

Ngb CDless crystals were obtained using the hanging drop vapor diffusion method in 0.8 M ammonium sulfate, 100 mM Bis-Tris pH 6.0 and 3% isopropanol, and the 2 μL-crystallization drop was composed of a mix of protein at 12 mg/mL and mother liquor in a 1:1 ratio. Crystals grew in approximately 2 months and were about 200 μm-long. Prior to data collection, crystals were cryo-protected in 20% glycerol-supplemented mother liquor, mounted on LithoLoops (Molecular Dimensions) of adequate size and flash-frozen in liquid nitrogen. X-ray diffraction data collection was performed at the I04 beamline (DIAMOND, United Kingdom). Ngb CDless crystals diffracted up to 1.80 Å. Preliminary data reduction (indexation, integration and scaling) was carried out by the DIAMOND on-site data analysis pipeline. The structure of Ngb CDless mutant was solved by molecular replacement (MOLREP, CCP4 7.0.078 release [1]) using the previously reported structure of Neuroglobin Gly-loop mutant (pdb code 6H6I [2]). Refinement

and model building were carried out using Refmac5 (CCP4 7.0.078 release [3]) or Phenix v1.14-3260 [4] and Coot 0.8.9.2 respectively [5]. Atomic coordinates of the ferric Ngb CDless structure were deposited in the Protein Data Bank under the accession number 7OHD. Images were prepared using Chimera 1.12 [6]. Structure determination statistics are reported in **Table S1**.

Resonance Raman Spectroscopy

The resonance Raman (RR) spectra of the crystals, mounted in capillaries, were obtained using a Renishaw RM2000 Raman microscope with a 514.5 nm line (Ar+ laser). The RR spectra of samples in solution in 100 mM HEPES pH 7.4, were obtained with excitation wavelengths a 514.5 (Ar+), 413.1 (Kr+), and 532 nm (diode laser Cobolt Samba 300). All the experimental conditions, including the experiments in polarized light, have been described previously[2,7]. The RR spectra were calibrated with indene, carbon tetrachloride and acetonitrile as standards to an accuracy of 1 cm−1 for intense isolated bands. All RR measurements were repeated several times to ensure reproducibility. To improve the signal-to-noise ratio, a number of spectra were accumulated and summed only if no spectral differences were noted. Absorption spectra were measured both prior to and after RR measurements to ensure that no degradation occurred under the experimental conditions used. All spectra were baseline corrected. The CO complexes have been obtained as reported by Exertier et al.[2]. However, since the CDless-CO complex appeared much more photolabile with respect to Ngb wild-type, the laser power was limited to 550 μW and a cylindrical lens was used to focus the beam on the sample. The curve-fitting analysis of the spectra was performed using a spectral simulation program (LabCalc; Fisher Scientific Company L.L.C., Pittsburg, PA, USA) with a Lorentzian line shape to determine the peak positions, bandwidths, and intensities.

Molecular dynamics simulations. The crystal unit cell was obtained by applying the P63 symmetry transformations to MON_1 and MON_2, two different murine neuroglobin CDless copies found in the asymmetric unit and described in the *Results and Discussion* section. The missing

residues were modeled by Molecular Operating Environment [8]. The unit cell was solvated with ~8000 TIP3P water molecules [9] and 48 Na^+ ions, with two successive solvent additions, each followed by a solvent relaxation session. The system was thermalized at 300 K in 10 ns, and equilibrated for 50 ns. A 500 ns-simulated tempering [10] simulation was then performed at a constant volume with the temperature ranging from 300 to 420 K in steps of 5 K. Starting coordinates of Ngb CDless in solution were taken from MON_1, one of the two monomers found in the asymmetric unit by X-ray crystallography. The protein was placed in a dodecahedron box with ~9000 TIP3P water molecules [9] and 4 Na^+ ions. After a thermalization of 10 ns and an equilibration run of 100 ns, simulated tempering simulations (1 μs) were done at a constant volume and with temperature ranging from 300 to 420 K in steps of 10 K. Pulling simulations [11] coupled with simulated tempering [10] were performed after breaking the bond between the heme and the distal histidine. The distance between heme iron and the distal histidine N_ε atom was the reaction coordinate. Representative configurations along the reaction coordinate were extracted, and 37 umbrella sampling simulations were spawned. Free energy profiles were obtained using the Weighted Histogram Analysis Method [12]. Starting coordinates of carboxy Ngb CDless in solution were generated from representative configurations of Ngb CDless with displaced distal histidine, after CO had been bound to the sixth coordination position. After the same equilibration procedure used for unliganded Ngb CDless, 300 ns-simulated tempering simulations [10] were performed. All the simulations were performed using GROMACS 2019.6 software package [13] and CHARMM36m force field [14]. The simulation protocols were the same as reported in [2].

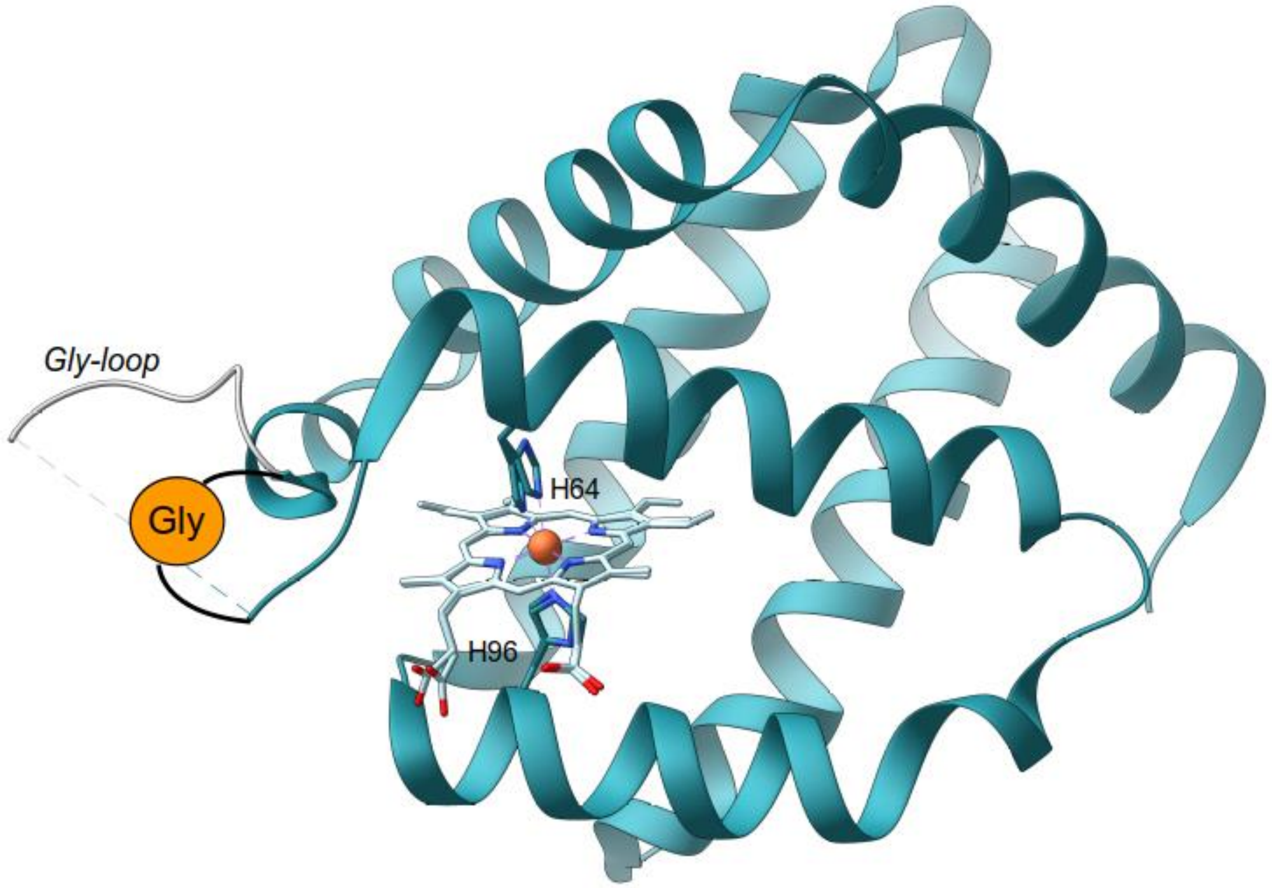

Figure S1. CDless mutant engineering on the basis of the structure of neuroglobin Gly-loop mutant (pdb code 6H6I [2]). The Gly-loop mutant was deprived of the Gln43-Leu56 segment to form the CDless mutant which contains a glycine residue between the C- and E-helix to form the so-called CElink.

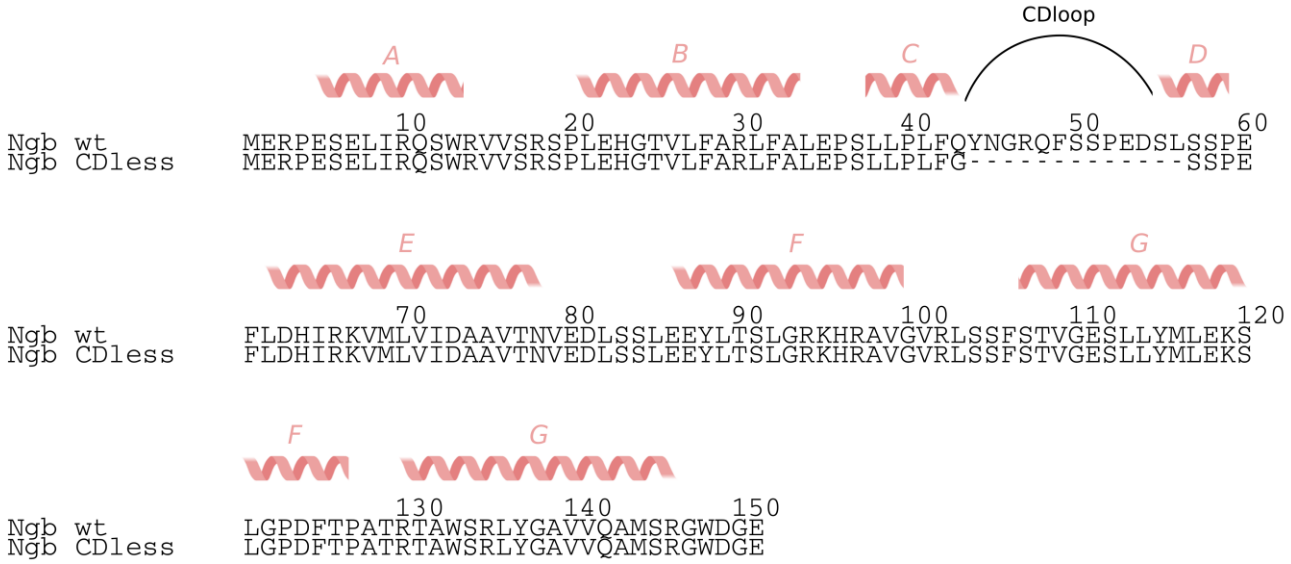

Figure S2. Sequence alignment of neuroglobin wild-type and CDless mutant. Helical secondary structures are represented in pink, while the CDloop originally present in the wild-type protein is indicated as a black arched line. Residues which were deleted in the CDless Ngb variant are represented as dashed lines. The sequence alignment was performed using ClustalW2 (EMBL-EBI).

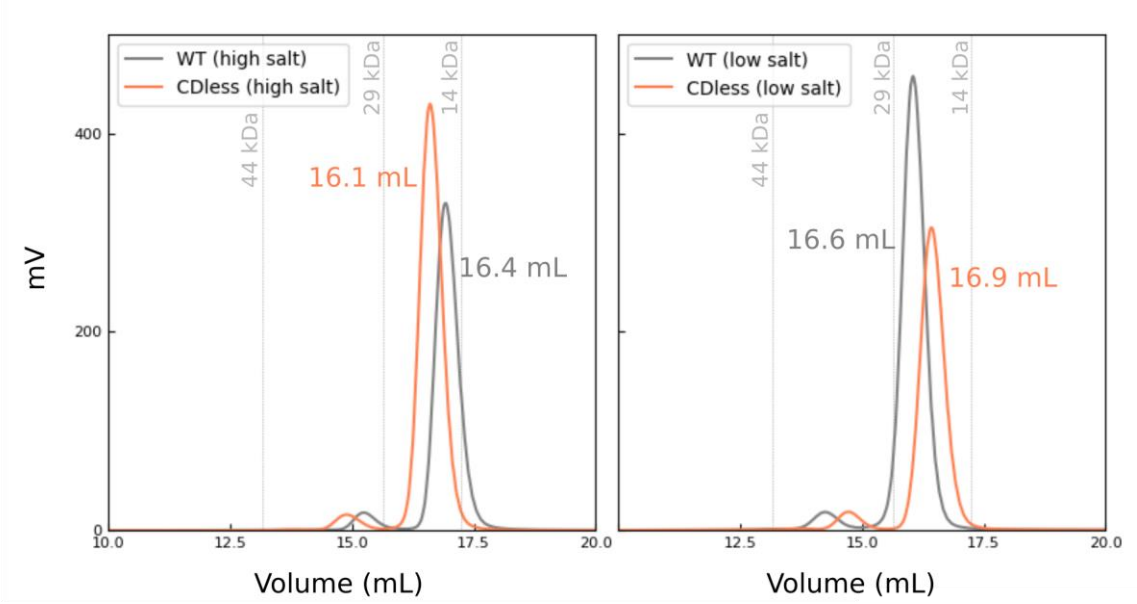

Figure S3. Oligomerization state of wild-type (WT) neuroglobin and the CDless mutant by size exclusion chromatography. Size exclusion chromatography was performed on a Cytiva LifeSciences Superdex 200 increase 10/300 GL coupled to a Knauer HPLC Azura pump system. The column was equilibrated either in 100 mM HEPES pH 7.4 (low salt) or in 100 mM HEPES pH 7.4, 200 mM NaCl (high salt). Prior to injection, samples were extensively centrifuged. Sample volumes of 200 uL of protein at 10 uM were injected onto the column at a flow rate of 0.5 mL/min. Protein elution was followed by absorption measurements at 412 nm (Soret). Molecular masses of protein peaks were estimated relative to those of standard proteins (GE Healthcare gel filtration calibration kit): ribonuclease A (14 kDa), carbonic anhydrase (29 kDa), ovalbumin (44 kDa). The wild-type and the CDless protein appears monomeric.

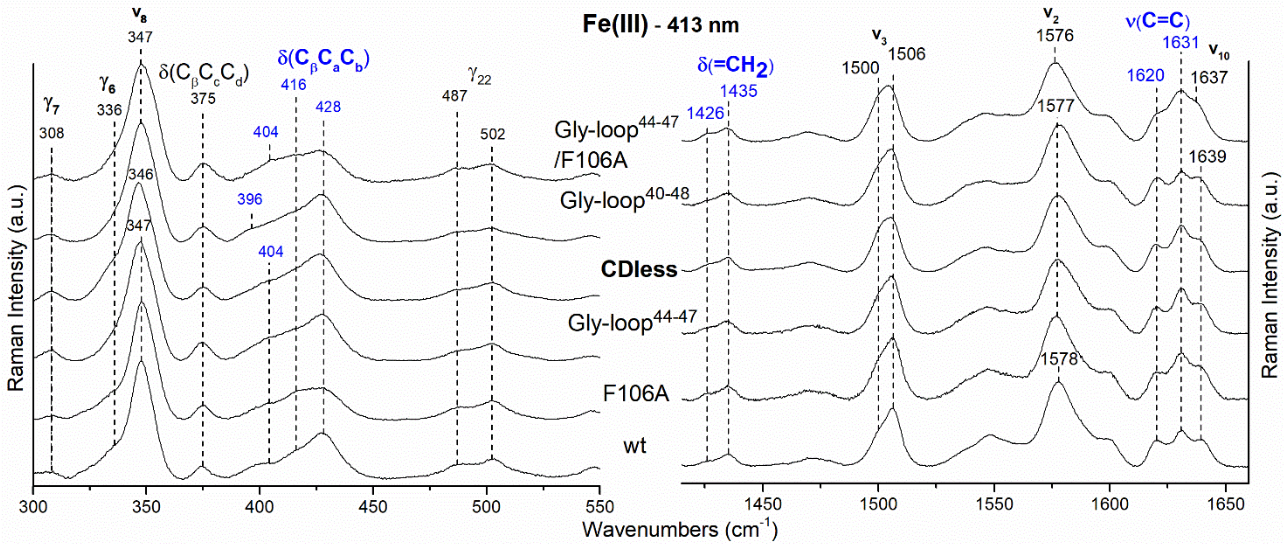

Figure S4: Comparison of the RR spectra in the high frequency (left) and low frequency (right) regions of the neuroglobin wild-type (WT) and mutants. Experimental conditions: 413.1 nm excitation wavelength, laser power at the sample 10 mW; WT: average of 6 spectra with 60 minutes integration time and 21 spectra with 210 minutes integration time for the high- and low-frequency regions, respectively; CDless: average of 20 spectra with 200 minutes integration time and 10 spectra with 100 minutes integration time for the high- and low-frequency regions, respectively; for the other mutants, see Ref. [7].

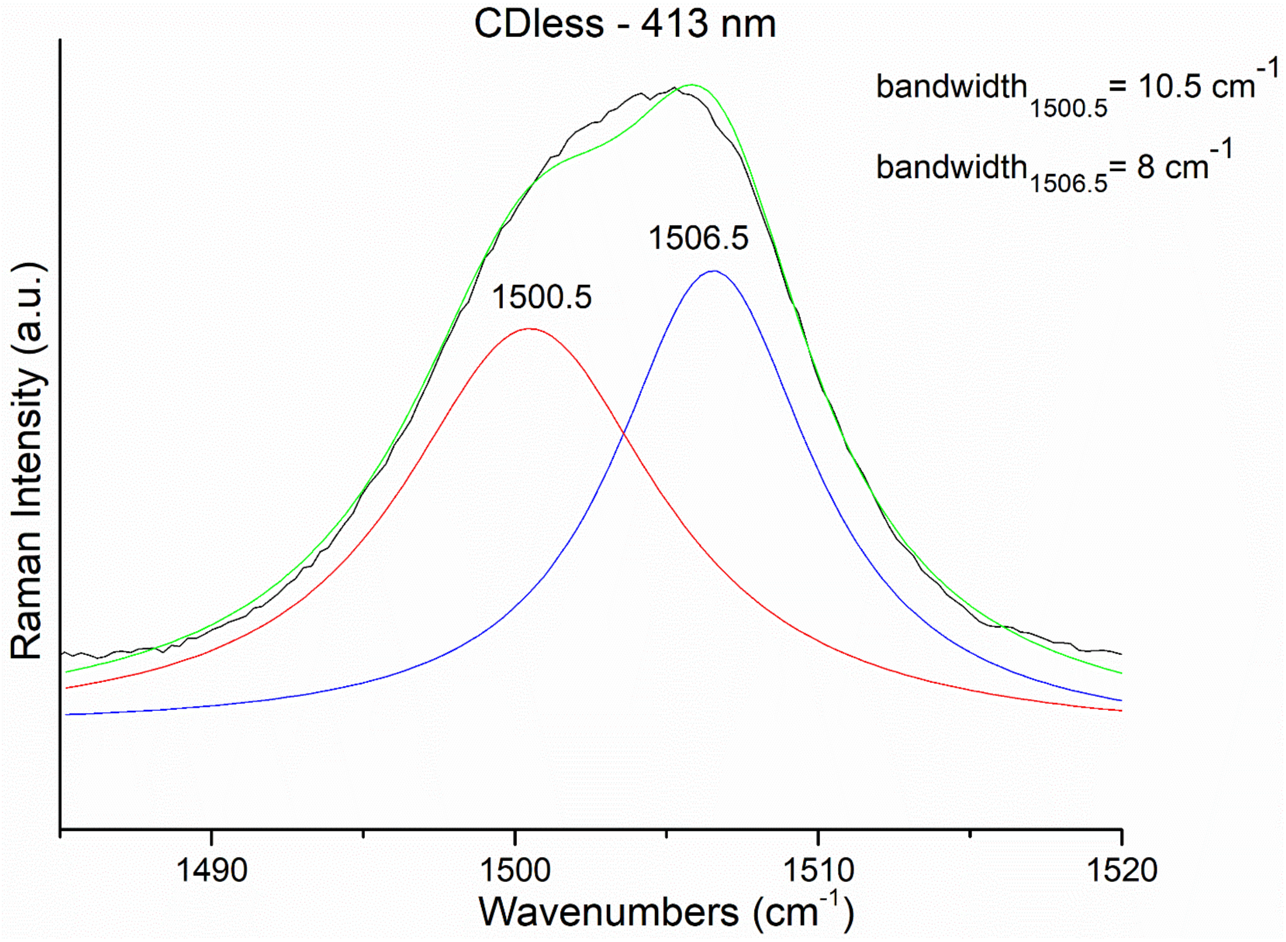

Figure S5. Curve-fitting analysis of the ν₃ region of the ferric CDless mutant: two bands model. The CDless spectrum region obtained with a 413.1 nm excitation wavelength curve-fitted with two ν₃ bands, using the same parameters of the wild-type (WT) in Figure 3B. Experimental conditions are the same as Figure S3.

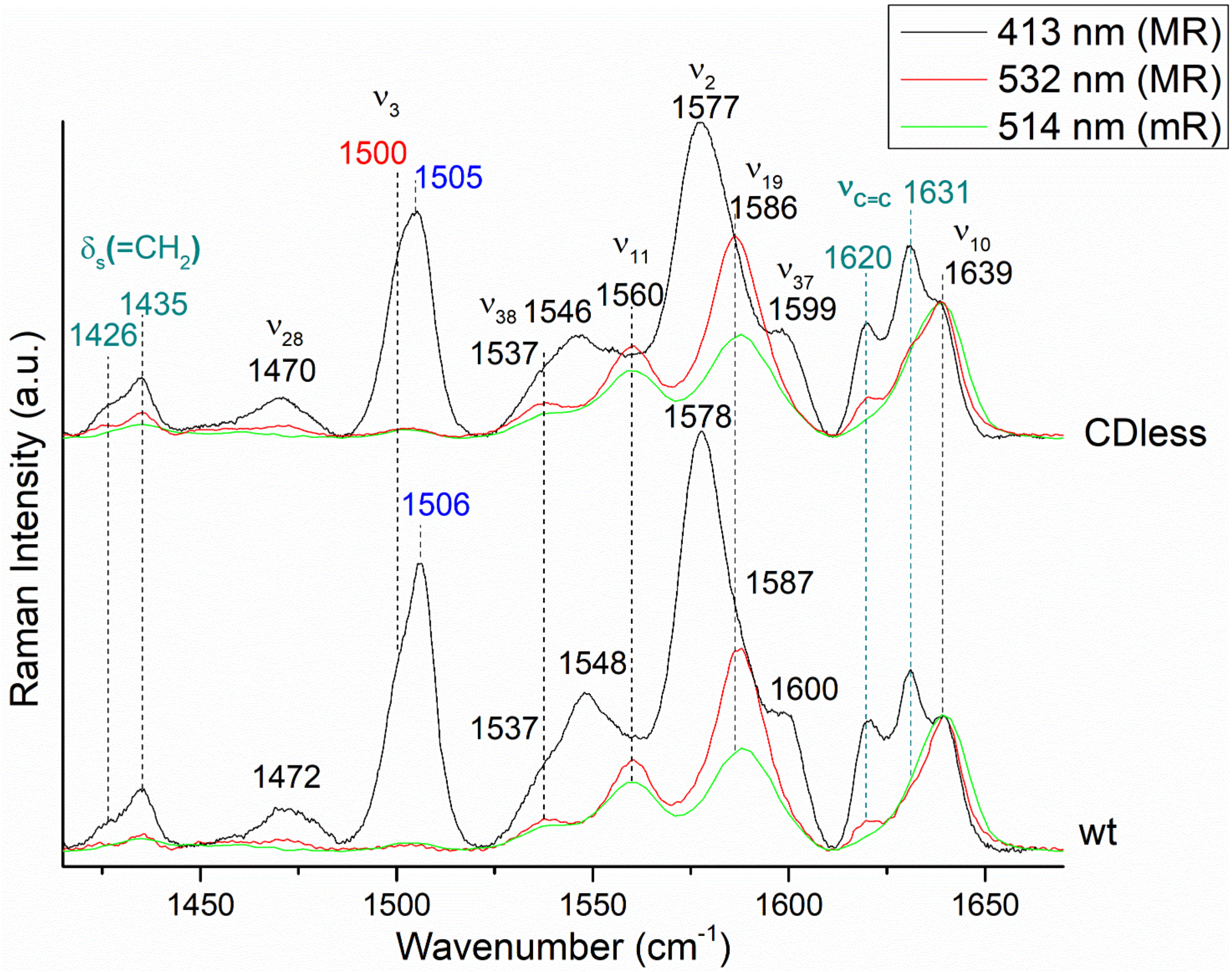

Figure S6. Comparison of the high frequency RR spectra of ferric Ngb wild-type (WT) and CDless mutant. The RR spectra were obtained with 413.1 (black line), 532 (red line) and 514.5 nm (green line) excitation wavelengths for the ferric Ngb CDless (top) and WT (bottom). The absence of any shift in the RR spectra obtained at λ_{exc} of 532 nm by the macroRaman (MR) spectrometer and at λ_{exc} of 514.5 nm by the microRaman (mR) spectrometer rules out any artifact due to the different set-ups and/or excitation wavelengths. The spectra have been shifted along the ordinate axis for better visualization. In the mutant the B conformer (ν_3 at 1500 cm^{-1}, red label) increases at the expense of the A form ((ν_3 at 1506 cm^{-1}, blue label). Experimental conditions: <u>413.1 nm excitation wavelength</u>, see Figure S3; <u>532 nm excitation wavelength</u>, laser power at the sample 30 mW; WT: average of 54 spectra with 270 minutes integration time; CDless: average of 44 spectra with 220 minutes integration time; <u>514.5 nm excitation wavelength</u>, laser power at the sample 2 mW; WT: average of 6 spectra with 30 minutes integration time; CDless: average of 6 spectra with 30 minutes integration time.

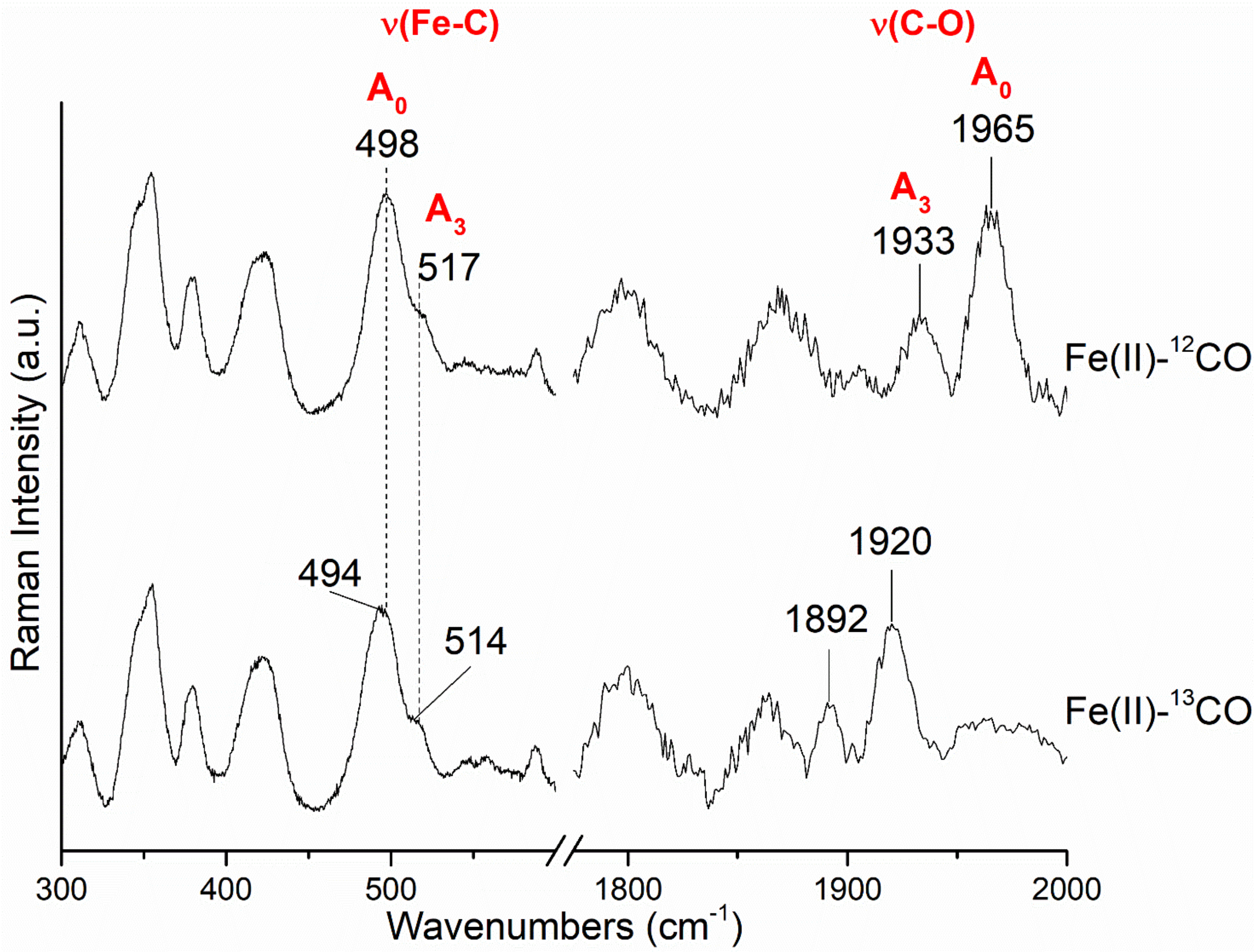

Figure S7. Fe(II)-CO complexes of Ngb CDless mutant. RR spectra in the low- (left) and high- (right) frequency regions of the Fe(II)-^{12}CO (top) and Fe(II)-^{13}CO (bottom) complexes. The frequencies of the ν(Fe-C), and ν(C-O) modes are labelled in red with the corresponding open (A_0) and closed (A_3) forms, in the low- and high-frequency regions, respectively. The spectra have been shifted along the ordinate axis to allow better visualization. Experimental conditions: λ_{exc} 413.1 nm, laser power at the sample 550 μW; total accumulation time for the ^{12}CO complex spectrum was 180–270 min and for the Fe(II)-^{13}CO complex spectrum 240–180 min in the low- and high-frequency regions, respectively.

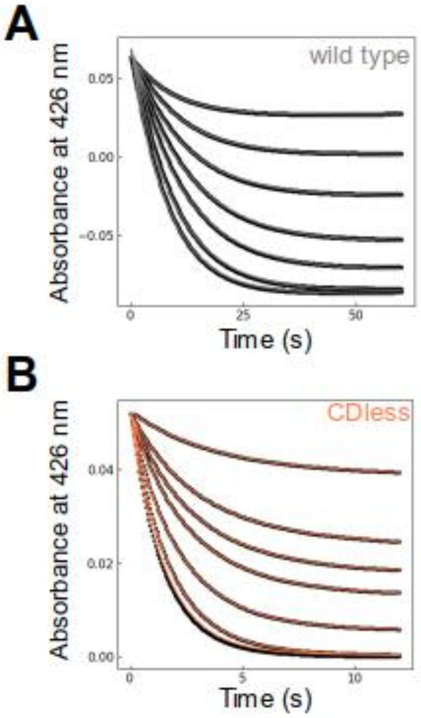

Figure S8. CO binding to neuroglobin wild-type and CDless mutant at 25°C by rapid mixing. CO binding to neuroglobin wild-type (**A**) and CDless (**B**) displays, respectively, a mono- and a bi-exponential decrease in absorbance at 426 nm. Experimental data are represented as black dots and the corresponding fits are shown in grey (Ngb wild-type) and orange (Ngb CDless).

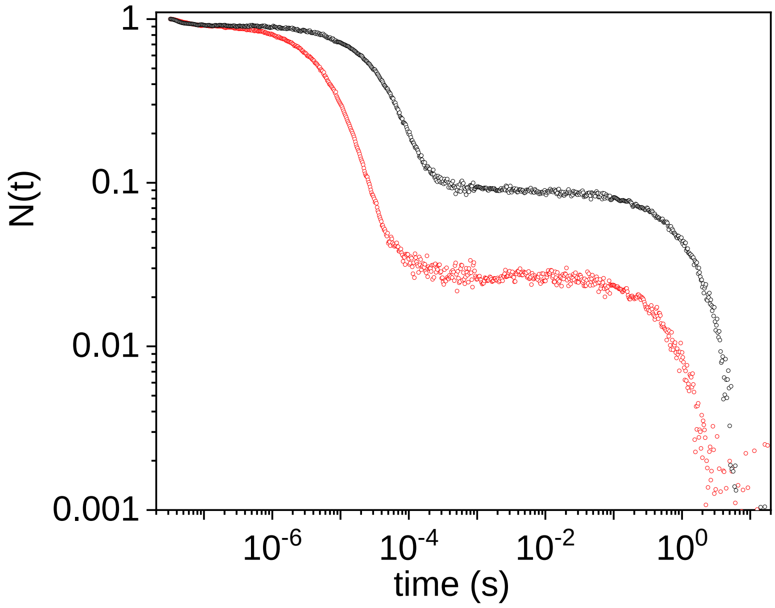

Figure S9: Representative CO rebinding kinetics to CDless mutant at 1 (red) and 0.2 (black) atm CO. Protein concentration was 44 μM, T=25 °C.

Identification of reaction steps

Figure S4 shows the CO rebinding kinetics after nanosecond laser photolysis at two different CO concentrations. Three main processes can be distinguished:

i) On the nanosecond time scale, an unimolecular process, independent on CO concentration, called geminate phase, that corresponds to the rebinding from the interior of the protein matrix, i.e. direct rebinding to the heme iron and migration to transient docking sites;

ii) On the microsecond time scale a bimolecular process, that speeds up upon increasing CO concentration, and that corresponds to rebinding of CO molecules from the solvent;

iii) A slower final reaction step, on the seconds time frame, that decreases in amplitude upon increasing CO concentration, and that correspond to the dissociation of the bis-histydil hexacoordinated complex.

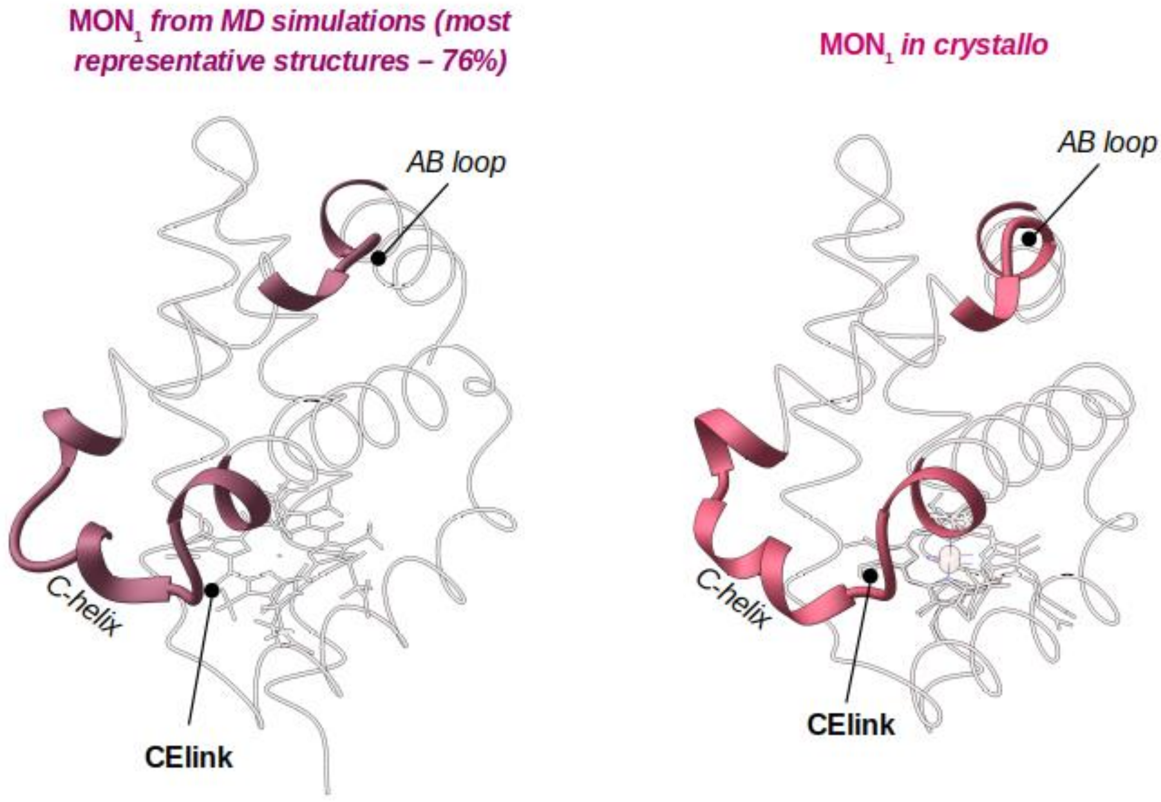

Figure S10. MON_1 most representative structures obtained by Molecular Dynamics simulations. The structure representing the most populated cluster (comprising 76% of the total structures) is displayed (left), with the heme depicted as colorless sticks. The heme is oriented according the most represented insertion observed in the cluster (59% of the structures with reversed insertion). As a comparison, the structure of MON_1 obtained by X-ray crystallography is reported on the right.

Structures are represented as ribbons, colored in purple and pink around the AB loop and CElink where the most significant differences are observed. Porphyrin rings are represented as colorless sticks.

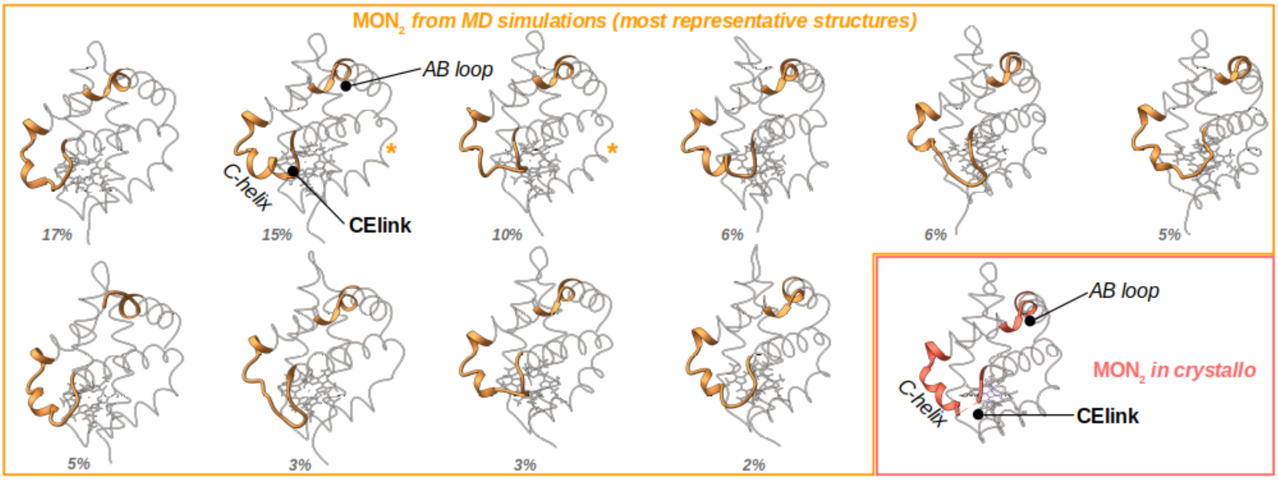

Figure S11. MON_2 most representative structures obtained by Molecular Dynamics simulations. The structures representing the ten most populated clusters, covering together 72% of the total structures, are displayed inside the orange frame. For each cluster, only the most represented heme insertion is displayed (in transparent sticks). Structures marked with an orange star embed the heme in canonical insertion. The canonical insertion represents in each cluster respectively 19%, 98%, 84%, 0%, 4%, 2%, 0%, 0%, 31%, 49% of the total heme insertions. As a comparison, the structure of MON_2 obtained by X-ray crystallography is reported inside the red frame. Structures are represented as ribbons, colored in orange and red around the AB loop and CElink where the most significant differences are observed. Porphyrin rings are represented as colorless sticks.

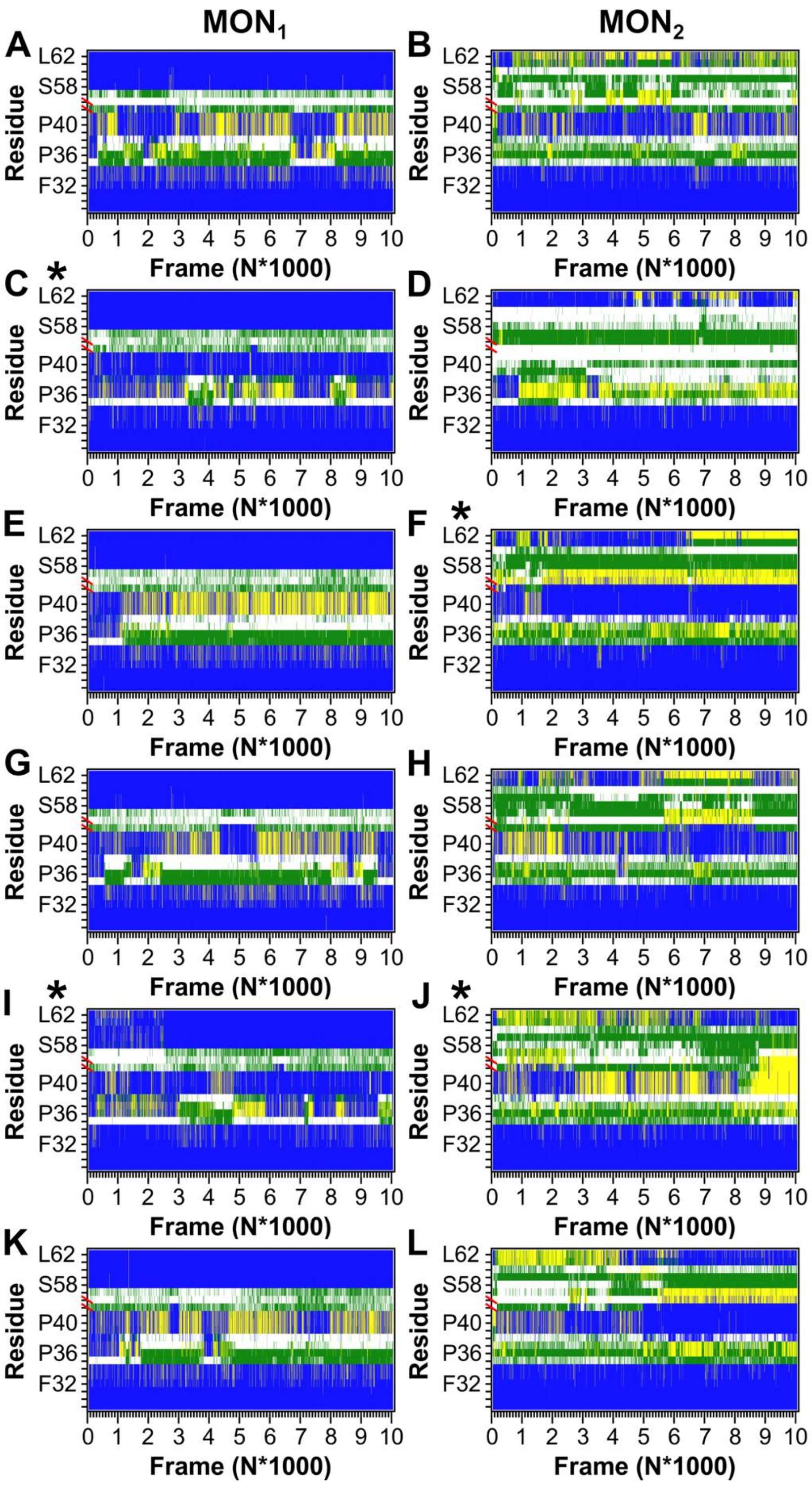

Figure S12. Stability of the C-helix for the twelve simulated structures of Ngb CDless in the unit cell by MD simulations. The original residue numeration from Ngb wild-type was maintained, and therefore the missing Q43-L56 sequence (*i.e.* CDless deletion) is represented by two red lines on the y-axis. Panels A, C, E, G, I and K represents the C-helix stability in MON_1 structure replicas, while panels B, D, F, H, J and L represents the C-helix stability in MON_2 structure replicas. Replicas with canonical heme insertion are marked with a star. Helices are represented in blue, coils in white, turns in yellow and bends in green.

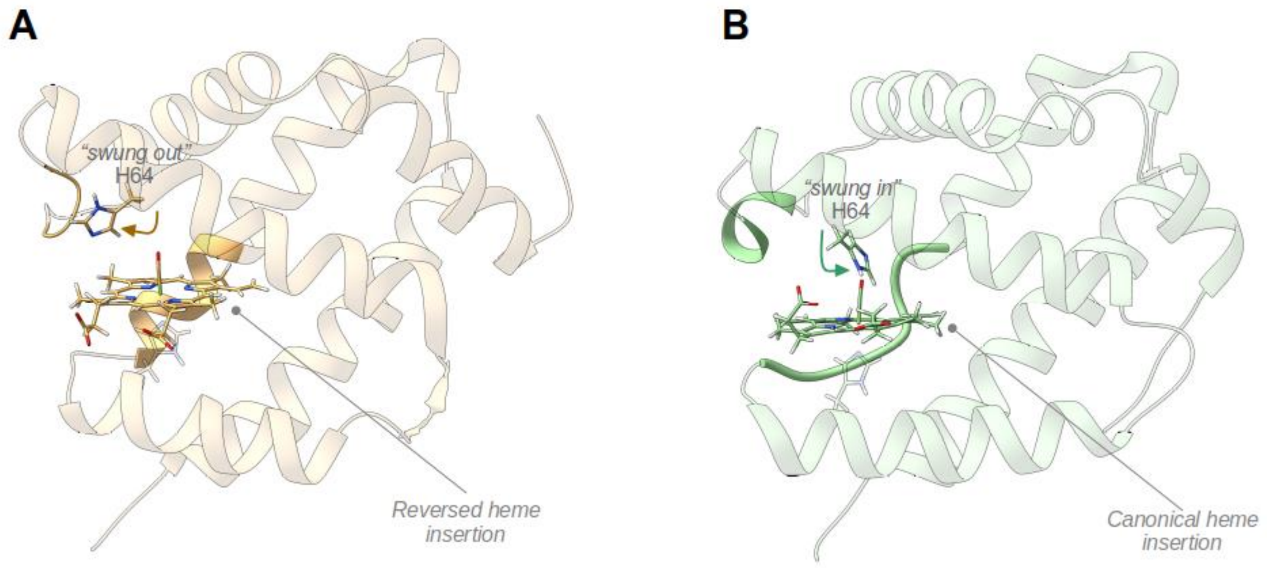

Figure S13. Influence of the heme insertion mode on His64 displacement and helix stability in murine Ngb by Molecular Dynamics simulations. The heme insertion affects the His64 displacement in carboxy Ngb CDless: in presence of the reversed heme insertion (**A**) His64 swings out, while in presence of the canonical insertion (**B**) His64 swings in. The heme insertion has also consequences on the secondary structure, as it affects the stability of the C-helix and of the N-terminus extremity of the G-helix. Secondary structures are displayed as ribbons. Residues, porphyrin groups and CO molecules are displayed as sticks.

Table S1. Crystallization, Data Collection, Refinements, Statistics and Validation. Statistics regarding the last resolution shell are reported into parenthesis**.** The R_{free} parameter is based on 5% of the data randomly selected.

**Data collection**	
PDB entry	7OHD
Number of images	3600
Space group	$P6_3$
Unit cell parameters (Å/°)	95.94/95.94/58.56 – 90/90/120
Resolution range (Å)	83.09 – 1.73 (1.93 – 1.73)
No of reflexions	570691 (97375)
No of unique reflections	30617 (7268)
Completeness (%)	100 (100)
I/sigma (I)	13.90 (0.98)
CC 1/2	100 (053.3)
Mosaicity	0.062
**Refinement**	
Resolution range (Å)	48.02 – 1.80
Wilson B-factors ($Å^2$)	47.71
Rwork/Rfree	0.182/0.211
Used Reflections	27161
**Number of atoms**	
Protein/Heme	2266/172
Sulfate ions/Bis-tris/Glycerol/Isopropanol	10/14/27/8

Water molecules	89
**Mean B factors (Å^2)**	
Protein/Heme/Solvent (Waters, others)	45.7/38.0/59.6
rmsd bond length (Å)	0.009
rmsd angles (°)	1.557
**Ramachandran plot statistics**	
Favored/Allowed/Outliers(%)	98.1/1.9/0

Table S2. Geometry of heme iron hexacoordination in neuroglobin CDless. Distances and angles were measured using coot 0.8.9.2

	WT	MON_1	MON_2
**His64-Fe in Å (reversed)**	1.9	2.0	2.1
**His96-Fe in Å (reversed)**	2.1	2.0	2.3
**His64-Fe-His96 angle (reversed)**	177.3°	163.3°	174.3°
**His64-Fe in Å (canonical)**	2.2	2.2	2.1
**His96-Fe in Å (canonical)**	1.9	2.2	1.9
**His64-Fe-His96 angle (canonical)**	157.5°	149.6°	173.0°

Table S3. Assignment of the RR bands. The frequencies (in cm^{-1}) of core size marker bands of the murine Ngb wild-type and CDless are reported and are indicated in blue for the reversed conformer A and in red for the canonical conformer B.

ν_{33}	B_{2g}	487	487
γ_{22}	E_g	502	502
ν_4	A_{1g}	1374	1374
$\delta(=CH_2)$		1426/1435	1426/1435
ν_{28}	B_{2g}	1472	1470
ν_3	A_{1g}	1500/1506	1500/1502/1506
ν_{38}	E_u	1537/1548	1537/1546
ν_{11}	B_{1g}	1560	1560
ν_2	A_{1g}	1575/1579	1575/1579
ν_{19}	A_{2g}	1587	1586
ν_{37}	E_u	1600	1599
$\nu(C=C)$		1620/1631	1620/1631
ν_{10}	B_{1g}	1637/1639	1637/1639

Table S4. ν(Fe-C) and ν(C-O) stretching mode frequencies of various Ngbs and swMb. The third weak H-bonded conformer (A_1), observed in human Ngb [19] and swMb [15] at 510/1948 and 508/1946 cm^{-1}, respectively, is not reported in the table.

	**(A_0) Open form No H-bond**		**(A_3) Closed form Strong H-bond**	
**Ngb**	ν(Fe-C)	ν(C-O)	ν(Fe-C)	ν(C-O)
C. aceratus [16]	489	1965	522	1934
D. mawsoni [16]	489	1965	522	1934
Mouse [17]	492	1969	523	1933
Human [18,19]	494	1972	521	1932
Murine WT [2]	493	1970	521	1933
Murine F106Av[2]	493	1970	519	1933
Murine Gly-loop [2]	495	1965	518	1933
Murine Gly-loop/F106A [2]	495	1970	518	1933
Murine CDless[This work]	498	1965	517	1933
**Mb** (Sperm Whale) [15]	493	1965	517	1932

Table S5. Lifetimes from the Fit of the Flash Photolysis Data at 25 °C and 0.2 atm CO. The relative amplitudes for the transients are reported in grey.

	Geminate phase	Geminate phase	Bimolecular phase 1	Bimolecular phase 2 + relaxation and His64 ligation	Relaxation and His64 ligation/ deligation	H64 deligation
	**τ1 (ns)**	**τ2 (ns)**	**τ3 (μs)**	**τ4 (μs)**	**τ5 (ms)**	**τ6 (s)**
**WT**	34 ± 1	-	83.2 ± 2	177 ± 8	34 ± 6	4.2 ± 0.4
	12%		55%	30%	1%	2%
**CDless**	18.3 ± 0.6	6.9 ± 0.4	46 ± 1	208 ± 24	175 ± 30	1.9 ± 0.1
	16%	7%	64%	5%	1%	7%

Table S6. Parameters retrieved from the fitting of the observed rate constant (k_{obs}) from rapid mixing experiments using equation S1. The parameters estimated from laser flash photolysis experiments are in grey in the table.

As shown in Figure 4A (main text) and S7A, kinetic traces from rapid mixing experiments can be fitted using an exponential decay function, yielding an estimate of the overall association rate constants (k_{obs}). For the wt mNgb, k_{obs} maintains a constant value, that allows the determination of k_{-H} only. For the other parameters, we report literature values for comparison. Regarding the CD-less mutant, k_{obs} increases upon increasing CO concentrations to a saturating limiting value.

The values of k_{obs} as a function of CO concentration can be analyzed using the following equation [22]:

$$k_{obs} = \frac{k_{-H} k_{on}[\mathrm{CO}]}{k_{-H}+k_{H}+k_{on}[\mathrm{CO}]} \quad \text{equation S1}$$

derived from the minimal kinetic scheme:

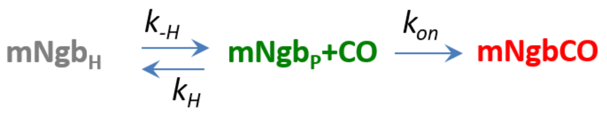

This procedure allows us to calculate mainly the distal histidine binding (k_H) and dissociation (k_{-H}) rate constants, and k_{on}. k_{on} and k_{-H} are in line with the values estimated by laser flash photolysis experiments.

	k_H (s^{-1})	k_{-H} (s^{-1})	k_{on} ($M^{-1}.s^{-1}$)
**WT**	1.0×10^3 [20]	0.50 [20]	7.27×10^7 [21]
**CDless** Fast phase	$4.9 \times 10^3 \pm 0.5$	1.2 ± 0.3	$1 \times 10^8 \pm 0.6$<br>1.1×10^8
**CDless** Slow phase	$4.2 \times 10^3 \pm 0.5$	0.68 ± 0.06<br>0.52	$6 \times 10^7 \pm 0.6$<br>2.4×10^7

References

(1) Vagin, A.; Teplyakov, A. Molecular Replacement with MOLREP. *Acta Crystallogr. Sect. D Biol. Crystallogr.* **2010**, *66* (1), 22–25. https://doi.org/10.1107/S0907444909042589.

(2) Exertier, C.; Milazzo, L.; Freda, I.; Montemiglio, L. C.; Scaglione, A.; Cerutti, G.; Parisi, G.; Anselmi, M.; Smulevich, G.; Savino, C. *et al*. Proximal and Distal Control for Ligand Binding in Neuroglobin: Role of the CD Loop and Evidence for His64 Gating. *Sci. Rep.* **2019**, *9* (1), 5326. https://doi.org/10.1038/s41598-019-41780-3.

(3) Murshudov, G. N.; Skubák, P.; Lebedev, A. A.; Pannu, N. S.; Steiner, R. A.; Nicholls, R. A.; Winn, M. D.; Long, F.; Vagin, A. A. REFMAC5 for the Refinement of Macromolecular Crystal Structures. *Acta Crystallogr. Sect. D Biol. Crystallogr.* **2011**, *67* (4), 355–367. https://doi.org/10.1107/S0907444911001314.

(4) Afonine, P. V.; Grosse-Kunstleve, R. W.; Echols, N.; Headd, J. J.; Moriarty, N. W.; Mustyakimov, M.; Terwilliger, T. C.; Urzhumtsev, A.; Zwart, P. H.; Adams, P. D. Towards Automated Crystallographic Structure Refinement with Phenix.Refine. *Acta Crystallogr. Sect. D Biol. Crystallogr.* **2012**, *68* (4), 352–367. https://doi.org/10.1107/S0907444912001308.

(5) Emsley, P.; Lohkamp, B.; Scott, W. G.; Cowtan, K. Features and Development of Coot. *Acta Crystallogr. Sect. D Biol. Crystallogr.* **2010**, *66* (4), 486–501. https://doi.org/10.1107/S0907444910007493.

(6) Pettersen, E. F.; Goddard, T. D.; Huang, C. C.; Couch, G. S.; Greenblatt, D. M.; Meng, E. C.; Ferrin, T. E. UCSF Chimera—A Visualization System for Exploratory Research and Analysis. *J Comput Chem* **2004**, *25*, 1605–1612. https://doi.org/10.1002/jcc.20084.

(7) Milazzo, L.; Exertier, C.; Becucci, M.; Freda, I.; Montemiglio, L. C.; Savino, C.; Vallone, B.; Smulevich, G. Lack of Orientation Selectivity of the Heme Insertion in Murine Neuroglobin Revealed by Resonance Raman Spectroscopy. *FEBS J.* **2020**, *287* (18), 4082–4097. https://doi.org/10.1111/febs.15241.

(8) Inc., C. C. G. Molecular Operating Environment (MOE), 2015.01. *1010 Sherbooke St.West, Suite #910, Montreal, QC, Canada, H3A 2R7*. 2015.

(9) Jorgensen, W. L.; Chandrasekhar, J.; Madura, J. D.; Impey, R. W.; Klein, M. L. Comparison of Simple Potential Functions for Simulating Liquid Water. *J. Chem. Phys.* **1983**, *79* (2), 926–935. https://doi.org/10.1063/1.445869.

(10) Marinari, E.; Parisi, G. Simulated Tempering: A New Monte Carlo Scheme. *EPL*. 1992, pp 451–458. https://doi.org/10.1209/0295-5075/19/6/002.

(11) Grubmüller, H. Force Probe Molecular Dynamics Simulations. *Methods Mol. Biol.* **2005**. https://doi.org/10.1007/978-1-59259-912-7_23.

(12) Hub, J. S.; de Groot, B. L.; van der Spoel, D. G_wham—A Free Weighted Histogram Analysis Implementation Including Robust Error and Autocorrelation Estimates. *J. Chem. Theory Comput.* **2010**, *6* (12), 3713–3720. https://doi.org/10.1021/ct100494z.

(13) Van Der Spoel, D.; Lindahl, E.; Hess, B.; Groenhof, G.; Mark, A. E.; Berendsen, H. J. C. GROMACS: Fast, Flexible, and Free. *J. Comput. Chem.* **2005**, *26* (16), 1701–1718. https://doi.org/10.1002/jcc.20291.

(14) Huang, J.; Rauscher, S.; Nawrocki, G.; Ran, T.; Feig, M.; De Groot, B. L.; Grubmüller, H.; MacKerell, A. D. CHARMM36m: An Improved Force Field for Folded and Intrinsically Disordered Proteins. *Nat. Methods* **2016**, *14* (1), 71–73. https://doi.org/10.1038/nmeth.4067.

(15) Morikis, D.; Champion, P. M.; Springer, B. A.; Sligar, S. G. Resonance Raman Investigations of Site-Directed Mutants of Myoglobin: Effects of Distal Histidine Replacement. *Biochemistry* **1989**, *28* (11), 4791–4800. https://doi.org/10.1021/bi00437a041.

(16) Giordano, D.; Boron, I.; Abbruzzetti, S.; Van Leuven, W.; Nicoletti, F. P.; Forti, F.; Bruno, S.; Cheng, C.-H. C.; Moens, L.; di Prisco, G. *et al*. Biophysical Characterisation of Neuroglobin of the Icefish, a Natural Knockout for Hemoglobin and Myoglobin. Comparison with Human Neuroglobin. *PLoS One* **2012**, *7* (12), e44508. https://doi.org/10.1371/journal.pone.0044508.

(17) Couture, M.; Burmester, T.; Hankeln, T.; Rousseau, D. L. The Heme Environment of Mouse Neuroglobin. Evidence for the Presence of Two Conformations of the Heme Pocket. *J. Biol. Chem.* **2001**, *276* (39), 36377–36382. https://doi.org/10.1074/jbc.M103907200.

(18) Ishikawa, H.; Finkelstein, I. J.; Kim, S.; Kwak, K.; Chung, J. K.; Wakasugi, K.; Massari, A. M.; Fayer, M. D. Neuroglobin Dynamics Observed with Ultrafast 2D-IR Vibrational Echo Spectroscopy. *Proc. Natl. Acad. Sci. U. S. A.* **2007**, *104* (41), 16116–16121. https://doi.org/10.1073/pnas.0707718104.

(19) Sawai, H.; Makino, M.; Mizutani, Y.; Ohta, T.; Sugimoto, H.; Uno, T.; Kawada, N.; Yoshizato, K.; Kitagawa, T.; Shiro, Y. Structural Characterization of the Proximal and Distal Histidine Environment of Cytoglobin and Neuroglobin. *Biochemistry* **2005**, *44* (40), 13257–13265. https://doi.org/10.1021/bi050997o.

(20) Kiger, L.; Uzan, J.; Dewilde, S.; Burmester, T.; Hankeln, T.; Moens, L.; Hamdane, D.; Baudin-Creuza, V.; Marden, M. Neuroglobin Ligand Binding Kinetics. *IUBMB Life* **2004**, *56* (11–12), 709–719. https://doi.org/10.1080/15216540500037711.

(21) Dewilde, S.; Kiger, L.; Burmester, T.; Hankeln, T.; Baudin-Creuza, V.; Aerts, T.; Marden, M. C.; Caubergs, R.; Moens, L. Biochemical Characterization and Ligand

Binding Properties of Neuroglobin, a Novel Member of the Globin Family. *J. Biol. Chem.* **2001**, *276* (42), 38949–38955. https://doi.org/10.1074/jbc.M106438200.

(22) Smagghe, B. J.; Sarath, G.; Ross, E.; Hilbert, J.; Hargrove, M. S. Slow Ligand Binding Kinetics Dominate Ferrous Hexacoordinate Hemoglobin Reactivities and Reveal Differences between Plants and Other Species †. *Biochemistry* **2006**, *45* (2), 561–570. https://doi.org/10.1021/bi051902l.
